# Supplementary material for: Design considerations for Factorial Adaptive Multi-Arm Multi-Stage (FAST) clinical trials
Source: Trials. 2024 Sep 12;25:608. doi: 10.1186/s13063-024-08400-6 (PMC11391813; doi:10.1186/s13063-024-08400-6)
Supplement: Supplementary file 1 — Supplementary Material 1. [file 13063_2024_8400_MOESM1_ESM.pdf]

Appendix A: Graphs

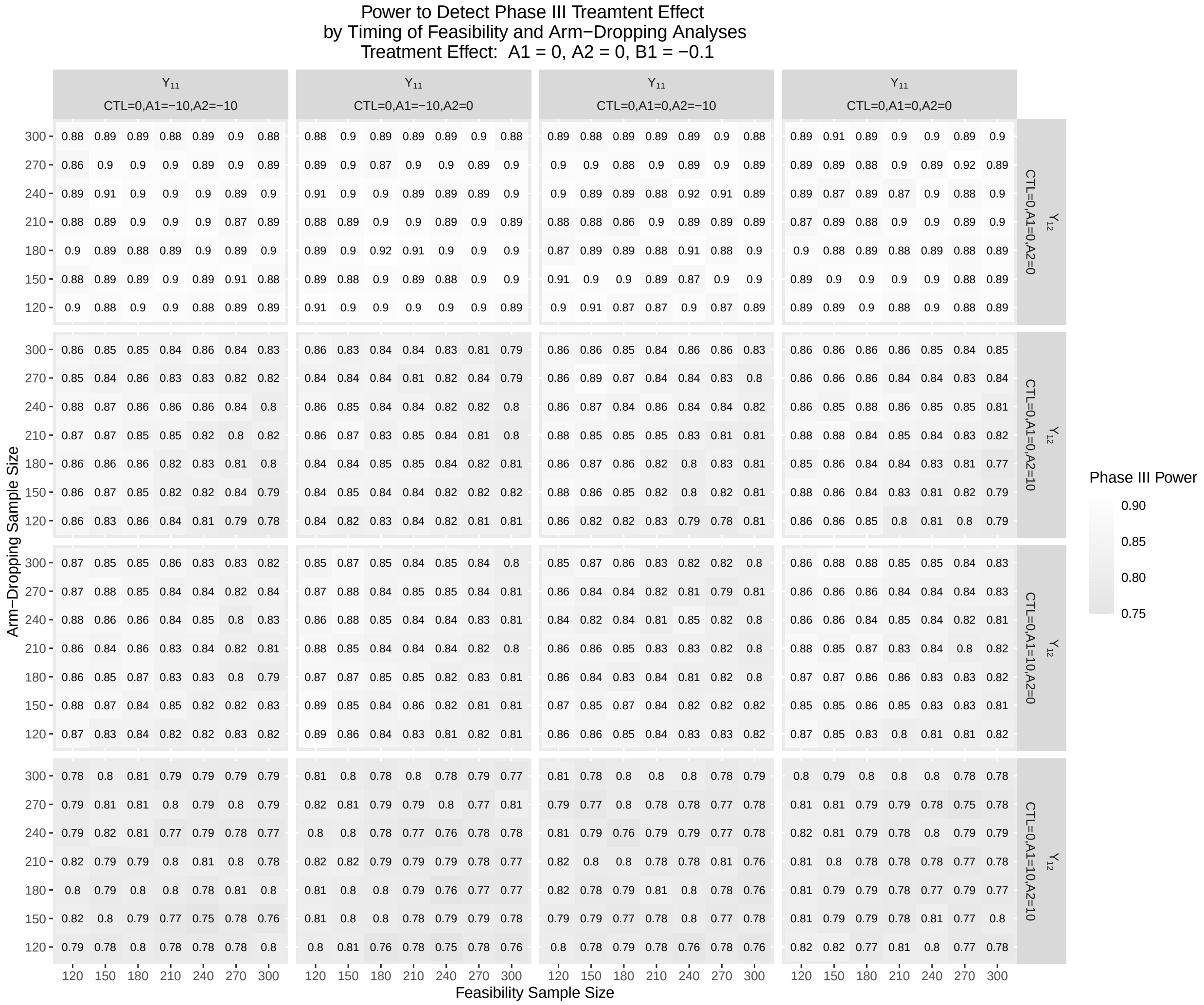

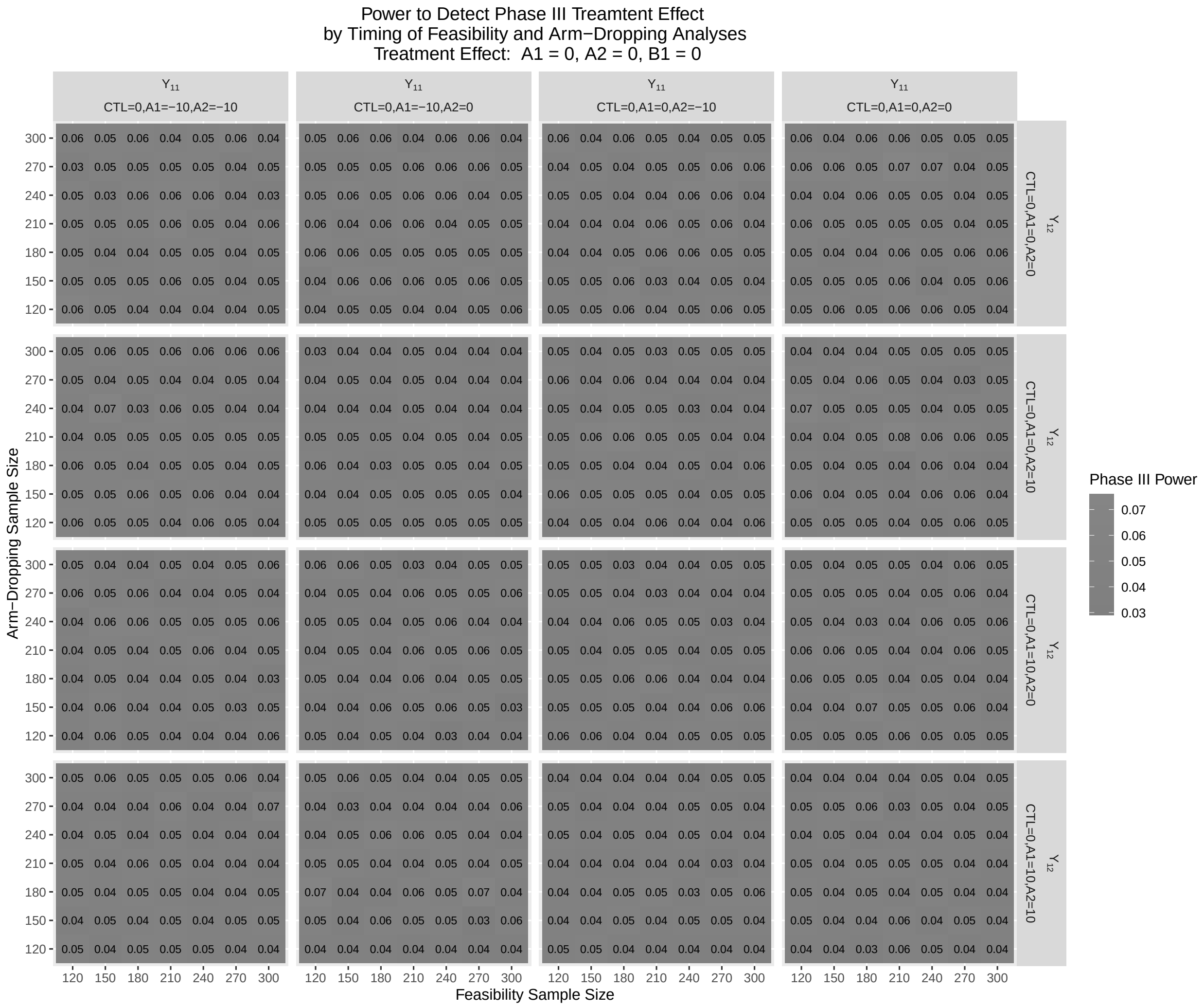
Power to Detect Phase III Treamtent Effect
by Timing of Feasibility and Arm−Dropping Analyses
Treatment Effect: A1 = 0, A2 = 0, B1 = 0
Y11
CTL=0,A1=−10,A2=−10
CTL=0,A1=−10,A2=0
CTL=0,A1=0,A2=−10
CTL=0,A1=0,A2=0
Y12
CTL=0,A1=0,A2=0
CTL=0,A1=0,A2=10
CTL=0,A1=10,A2=0
CTL=0,A1=10,A2=10
Arm−Dropping Sample Size
Feasibility Sample Size
120 150 180 210 240 270 300
Phase III Power
0.07
0.06
0.05
0.04
0.03

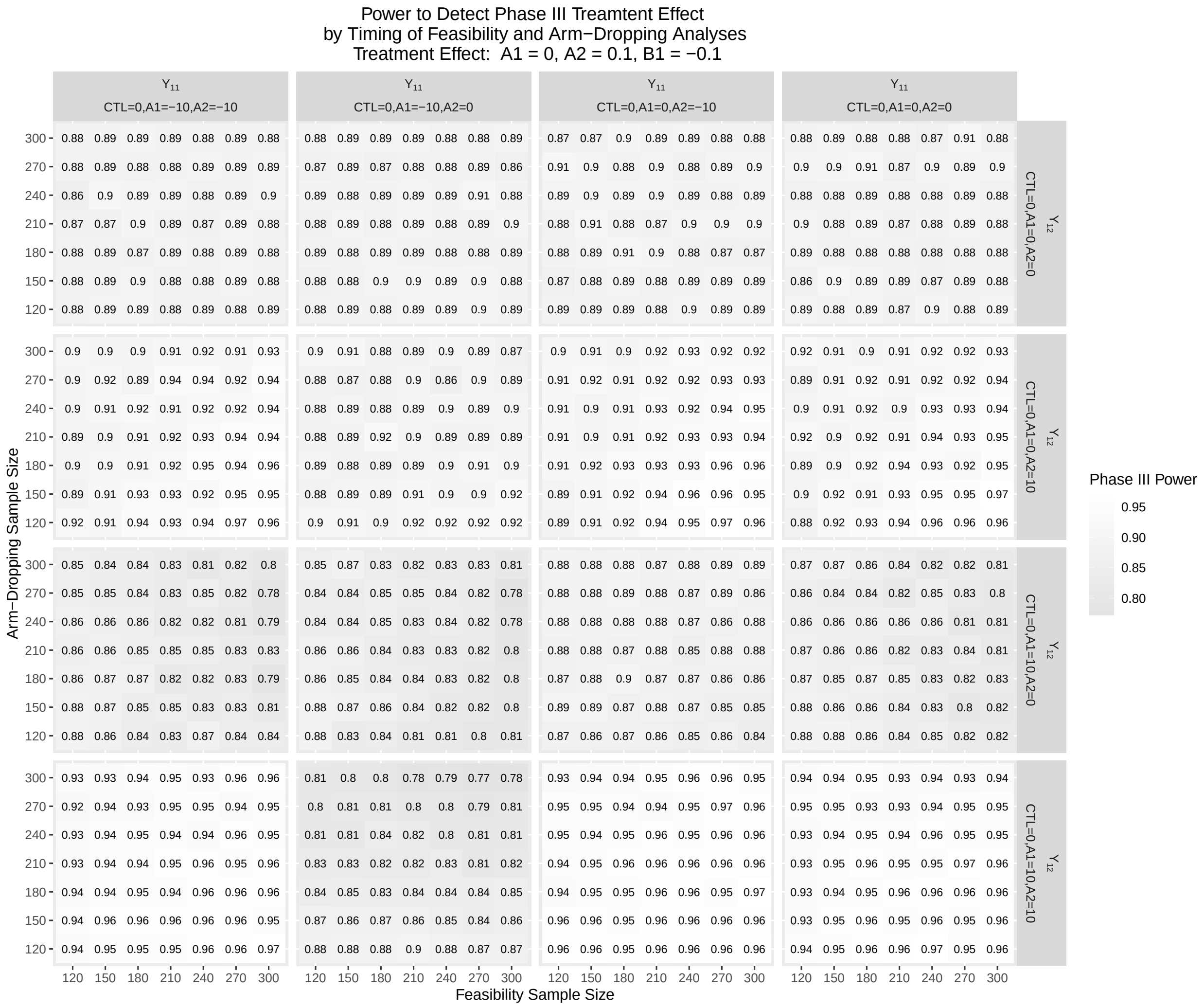
Power to Detect Phase III Treamtent Effect
by Timing of Feasibility and Arm−Dropping Analyses
Treatment Effect: A1 = 0, A2 = 0.1, B1 = −0.1
Y11 CTL=0,A1=−10,A2=−10
Y11 CTL=0,A1=−10,A2=0
Y11 CTL=0,A1=0,A2=−10
Y11 CTL=0,A1=0,A2=0
Y12 CTL=0,A1=0,A2=0
Y12 CTL=0,A1=0,A2=10
Y12 CTL=0,A1=10,A2=0
Y12 CTL=0,A1=10,A2=10
Arm−Dropping Sample Size
Feasibility Sample Size
Phase III Power
0.95
0.90
0.85
0.80

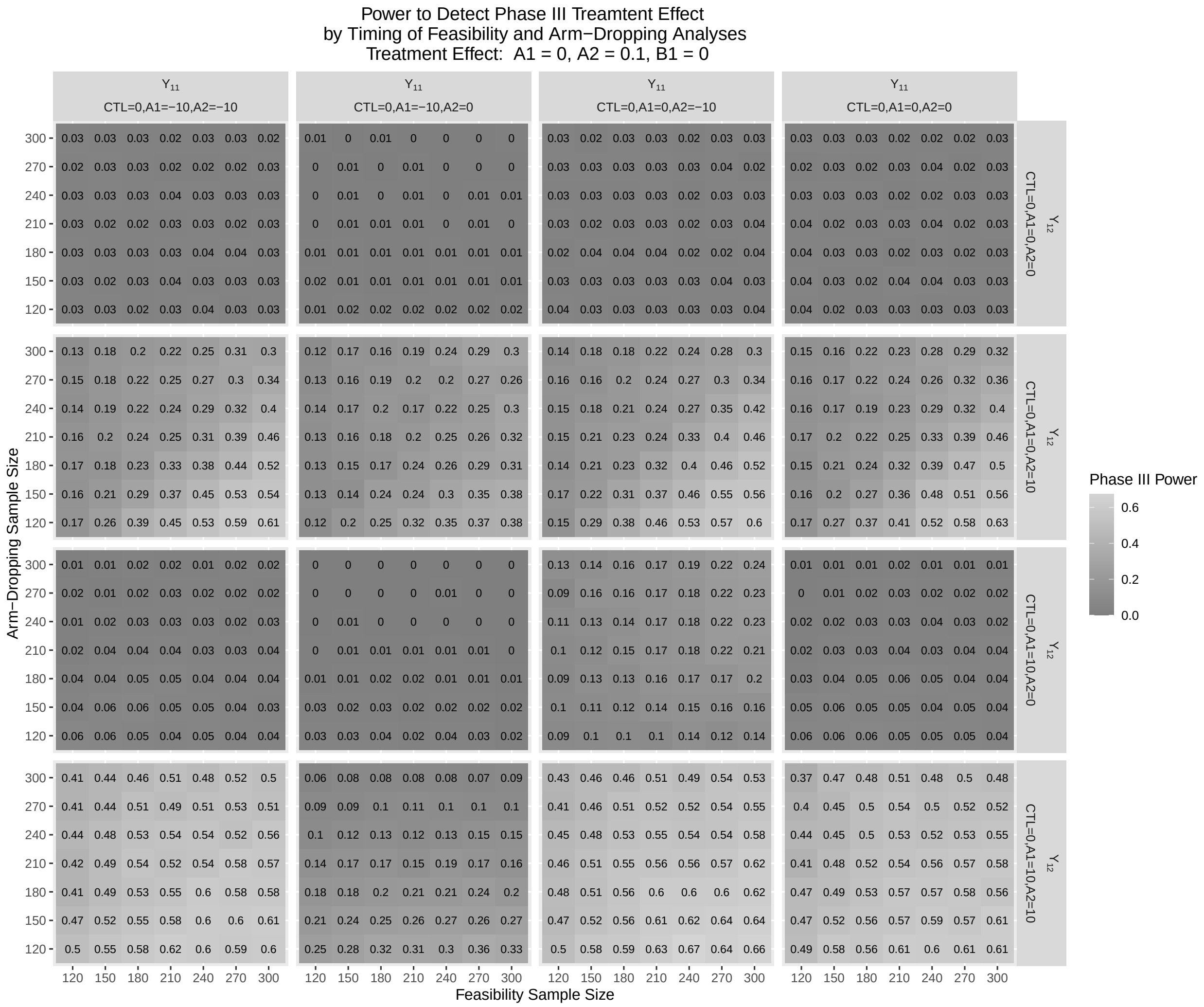
Power to Detect Phase III Treamtent Effect
by Timing of Feasibility and Arm−Dropping Analyses
Treatment Effect: A1 = 0, A2 = 0.1, B1 = 0
Y_{11} CTL=0,A1=−10,A2=−10
Y_{11} CTL=0,A1=−10,A2=0
Y_{11} CTL=0,A1=0,A2=−10
Y_{11} CTL=0,A1=0,A2=0
Y_{12} CTL=0,A1=0,A2=0
Y_{12} CTL=0,A1=0,A2=10
Y_{12} CTL=0,A1=10,A2=0
Y_{12} CTL=0,A1=10,A2=10
Arm−Dropping Sample Size
Feasibility Sample Size
Phase III Power
0.0
0.2
0.4
0.6

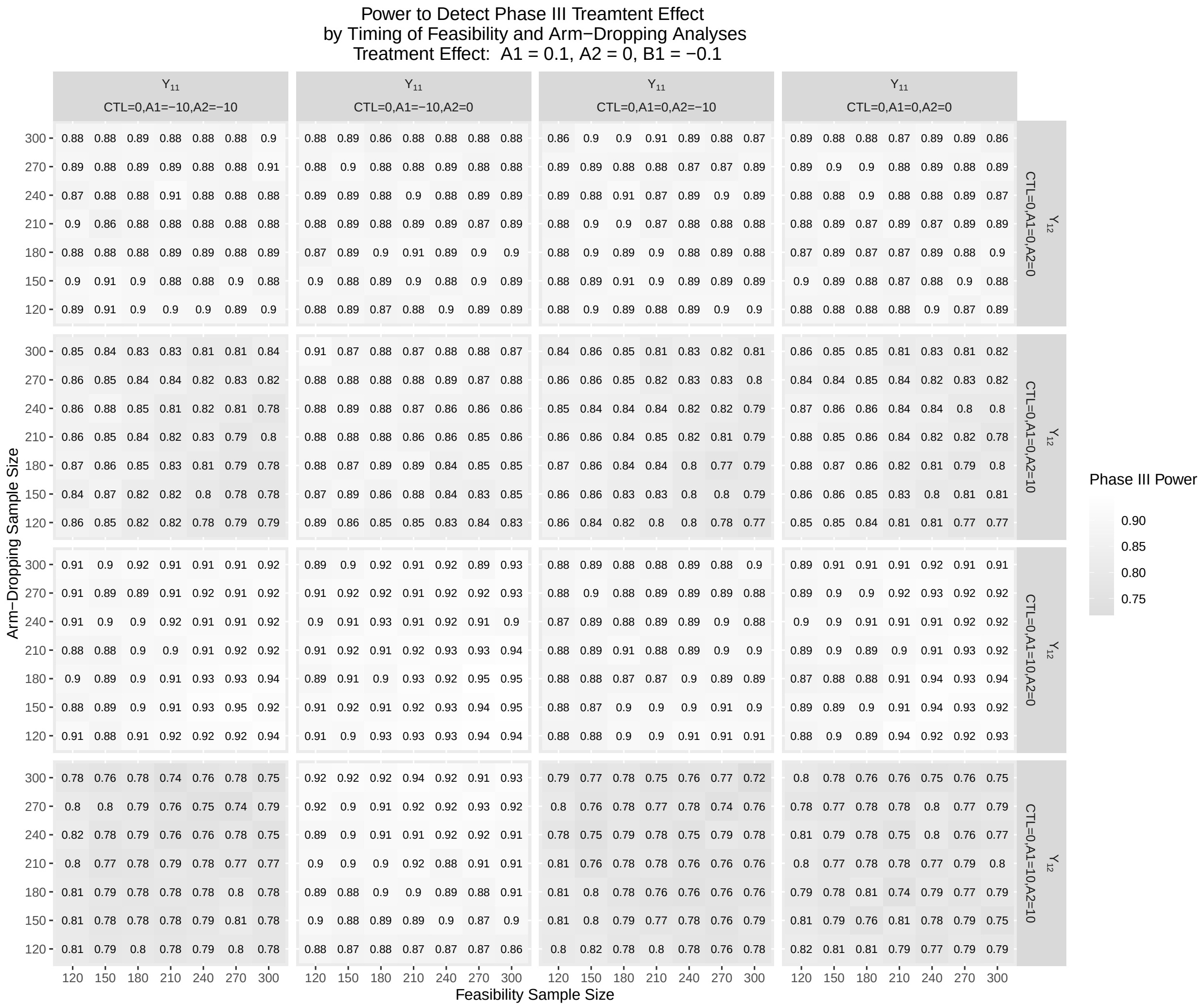

Power to Detect Phase III Treamtent Effect
by Timing of Feasibility and Arm−Dropping Analyses
Treatment Effect: A1 = 0.1, A2 = 0, B1 = −0.1
Y11 CTL=0,A1=−10,A2=−10 | Y11 CTL=0,A1=−10,A2=0 | Y11 CTL=0,A1=0,A2=−10 | Y11 CTL=0,A1=0,A2=0
Y12 CTL=0,A1=0,A2=0
300 0.88 0.88 0.89 0.88 0.88 0.88 0.9 | 0.88 0.89 0.86 0.88 0.88 0.88 0.88 | 0.86 0.9 0.9 0.91 0.89 0.88 0.87 | 0.89 0.88 0.88 0.87 0.89 0.89 0.86
270 0.89 0.88 0.89 0.89 0.88 0.88 0.91 | 0.88 0.9 0.88 0.88 0.89 0.88 0.88 | 0.89 0.89 0.88 0.88 0.87 0.87 0.89 | 0.89 0.9 0.9 0.88 0.89 0.88 0.89
240 0.87 0.88 0.88 0.91 0.88 0.88 0.88 | 0.89 0.89 0.88 0.9 0.88 0.89 0.89 | 0.89 0.88 0.91 0.87 0.89 0.9 0.89 | 0.88 0.88 0.9 0.88 0.88 0.89 0.87
210 0.9 0.86 0.88 0.88 0.88 0.88 0.88 | 0.88 0.89 0.88 0.89 0.89 0.87 0.89 | 0.88 0.9 0.9 0.87 0.88 0.88 0.88 | 0.88 0.89 0.87 0.89 0.87 0.89 0.89
180 0.88 0.88 0.88 0.89 0.89 0.88 0.89 | 0.87 0.89 0.9 0.91 0.89 0.9 0.9 | 0.88 0.9 0.89 0.9 0.88 0.89 0.88 | 0.87 0.89 0.87 0.87 0.89 0.88 0.9
150 0.9 0.91 0.9 0.88 0.88 0.9 0.88 | 0.9 0.88 0.89 0.9 0.88 0.9 0.89 | 0.88 0.89 0.91 0.9 0.89 0.89 0.89 | 0.9 0.89 0.88 0.87 0.88 0.9 0.88
120 0.89 0.91 0.9 0.9 0.9 0.89 0.9 | 0.88 0.89 0.87 0.88 0.9 0.89 0.89 | 0.88 0.9 0.89 0.88 0.89 0.9 0.9 | 0.88 0.88 0.88 0.88 0.9 0.87 0.89
Y12 CTL=0,A1=0,A2=10
300 0.85 0.84 0.83 0.83 0.81 0.81 0.84 | 0.91 0.87 0.88 0.87 0.88 0.88 0.87 | 0.84 0.86 0.85 0.81 0.83 0.82 0.81 | 0.86 0.85 0.85 0.81 0.83 0.81 0.82
270 0.86 0.85 0.84 0.84 0.82 0.83 0.82 | 0.88 0.88 0.88 0.88 0.89 0.87 0.88 | 0.86 0.86 0.85 0.82 0.83 0.83 0.8 | 0.84 0.84 0.85 0.84 0.82 0.83 0.82
240 0.86 0.88 0.85 0.81 0.82 0.81 0.78 | 0.88 0.89 0.88 0.87 0.86 0.86 0.86 | 0.85 0.84 0.84 0.84 0.82 0.82 0.79 | 0.87 0.86 0.86 0.84 0.84 0.8 0.8
210 0.86 0.85 0.84 0.82 0.83 0.79 0.8 | 0.88 0.88 0.88 0.86 0.86 0.85 0.86 | 0.86 0.86 0.84 0.85 0.82 0.81 0.79 | 0.88 0.85 0.86 0.84 0.82 0.82 0.78
180 0.87 0.86 0.85 0.83 0.81 0.79 0.78 | 0.88 0.87 0.89 0.89 0.84 0.85 0.85 | 0.87 0.86 0.84 0.84 0.8 0.77 0.79 | 0.88 0.87 0.86 0.82 0.81 0.79 0.8
150 0.84 0.87 0.82 0.82 0.8 0.78 0.78 | 0.87 0.89 0.86 0.88 0.84 0.83 0.85 | 0.86 0.86 0.83 0.83 0.8 0.8 0.79 | 0.86 0.86 0.85 0.83 0.8 0.81 0.81
120 0.86 0.85 0.82 0.82 0.78 0.79 0.79 | 0.89 0.86 0.85 0.85 0.83 0.84 0.83 | 0.86 0.84 0.82 0.8 0.8 0.78 0.77 | 0.85 0.85 0.84 0.81 0.81 0.77 0.77
Y12 CTL=0,A1=10,A2=0
300 0.91 0.9 0.92 0.91 0.91 0.91 0.92 | 0.89 0.9 0.92 0.91 0.92 0.89 0.93 | 0.88 0.89 0.88 0.88 0.89 0.88 0.9 | 0.89 0.91 0.91 0.91 0.92 0.91 0.91
270 0.91 0.89 0.89 0.91 0.92 0.91 0.92 | 0.91 0.92 0.92 0.91 0.92 0.92 0.93 | 0.88 0.9 0.88 0.89 0.89 0.89 0.88 | 0.89 0.9 0.9 0.92 0.93 0.92 0.92
240 0.91 0.9 0.9 0.92 0.91 0.91 0.92 | 0.9 0.91 0.93 0.91 0.92 0.91 0.9 | 0.87 0.89 0.88 0.89 0.89 0.9 0.88 | 0.9 0.9 0.91 0.91 0.91 0.92 0.92
210 0.88 0.88 0.9 0.9 0.91 0.92 0.92 | 0.91 0.92 0.91 0.92 0.93 0.93 0.94 | 0.88 0.89 0.91 0.88 0.89 0.9 0.9 | 0.89 0.9 0.89 0.9 0.91 0.93 0.92
180 0.9 0.89 0.9 0.91 0.93 0.93 0.94 | 0.89 0.91 0.9 0.93 0.92 0.95 0.95 | 0.88 0.88 0.87 0.87 0.9 0.89 0.89 | 0.87 0.88 0.88 0.91 0.94 0.93 0.94
150 0.88 0.89 0.9 0.91 0.93 0.95 0.92 | 0.91 0.92 0.91 0.92 0.93 0.94 0.95 | 0.88 0.87 0.9 0.9 0.9 0.91 0.9 | 0.89 0.89 0.9 0.91 0.94 0.93 0.92
120 0.91 0.88 0.91 0.92 0.92 0.92 0.94 | 0.91 0.9 0.93 0.93 0.93 0.94 0.94 | 0.88 0.88 0.9 0.9 0.91 0.91 0.91 | 0.88 0.9 0.89 0.94 0.92 0.92 0.93
Y12 CTL=0,A1=10,A2=10
300 0.78 0.76 0.78 0.74 0.76 0.78 0.75 | 0.92 0.92 0.92 0.94 0.92 0.91 0.93 | 0.79 0.77 0.78 0.75 0.76 0.77 0.72 | 0.8 0.78 0.76 0.76 0.75 0.76 0.75
270 0.8 0.8 0.79 0.76 0.75 0.74 0.79 | 0.92 0.9 0.91 0.92 0.92 0.93 0.92 | 0.8 0.76 0.78 0.77 0.78 0.74 0.76 | 0.78 0.77 0.78 0.78 0.8 0.77 0.79
240 0.82 0.78 0.79 0.76 0.76 0.78 0.75 | 0.89 0.9 0.91 0.91 0.92 0.92 0.91 | 0.78 0.75 0.79 0.78 0.75 0.79 0.78 | 0.81 0.79 0.78 0.75 0.8 0.76 0.77
210 0.8 0.77 0.78 0.79 0.78 0.77 0.77 | 0.9 0.9 0.9 0.92 0.88 0.91 0.91 | 0.81 0.76 0.78 0.78 0.76 0.76 0.76 | 0.8 0.77 0.78 0.78 0.77 0.79 0.8
180 0.81 0.79 0.78 0.78 0.78 0.8 0.78 | 0.89 0.88 0.9 0.9 0.89 0.88 0.91 | 0.81 0.8 0.78 0.76 0.76 0.76 0.76 | 0.79 0.78 0.81 0.74 0.79 0.77 0.79
150 0.81 0.78 0.78 0.78 0.79 0.81 0.78 | 0.9 0.88 0.89 0.89 0.9 0.87 0.9 | 0.81 0.8 0.79 0.77 0.78 0.76 0.79 | 0.81 0.79 0.76 0.81 0.78 0.79 0.75
120 0.81 0.79 0.8 0.78 0.79 0.8 0.78 | 0.88 0.87 0.88 0.87 0.87 0.87 0.86 | 0.8 0.82 0.78 0.8 0.78 0.76 0.78 | 0.82 0.81 0.81 0.79 0.77 0.79 0.79
120 150 180 210 240 270 300 | 120 150 180 210 240 270 300 | 120 150 180 210 240 270 300 | 120 150 180 210 240 270 300
Feasibility Sample Size
Arm−Dropping Sample Size
Phase III Power
0.90
0.85
0.80
0.75

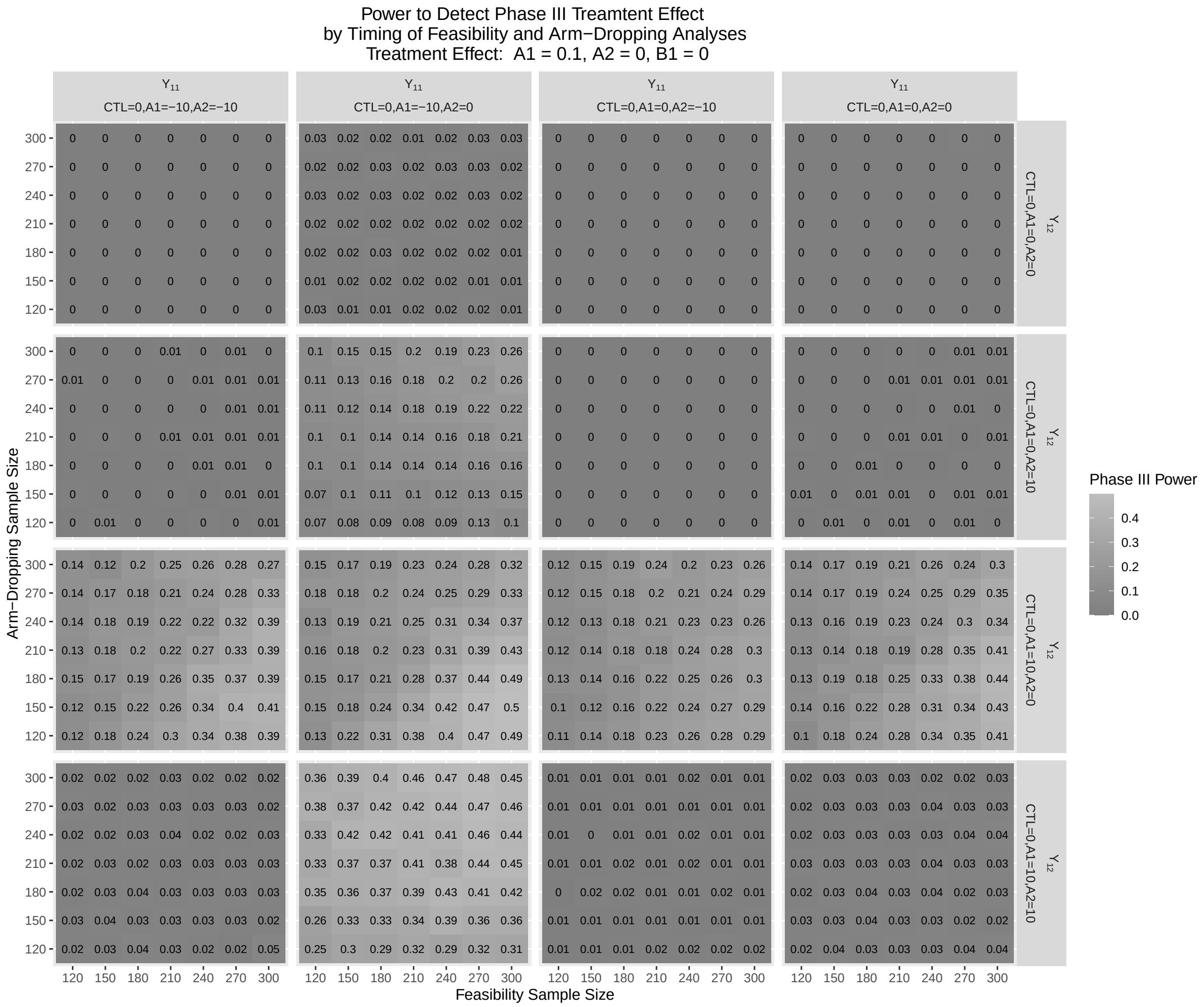
Power to Detect Phase III Treamtent Effect
by Timing of Feasibility and Arm−Dropping Analyses
Treatment Effect: A1 = 0.1, A2 = 0, B1 = 0
Y11
CTL=0,A1=−10,A2=−10
CTL=0,A1=−10,A2=0
CTL=0,A1=0,A2=−10
CTL=0,A1=0,A2=0
Y12
CTL=0,A1=0,A2=0
CTL=0,A1=0,A2=10
CTL=0,A1=10,A2=0
CTL=0,A1=10,A2=10
Arm−Dropping Sample Size
Feasibility Sample Size
Phase III Power
0.4
0.3
0.2
0.1
0.0

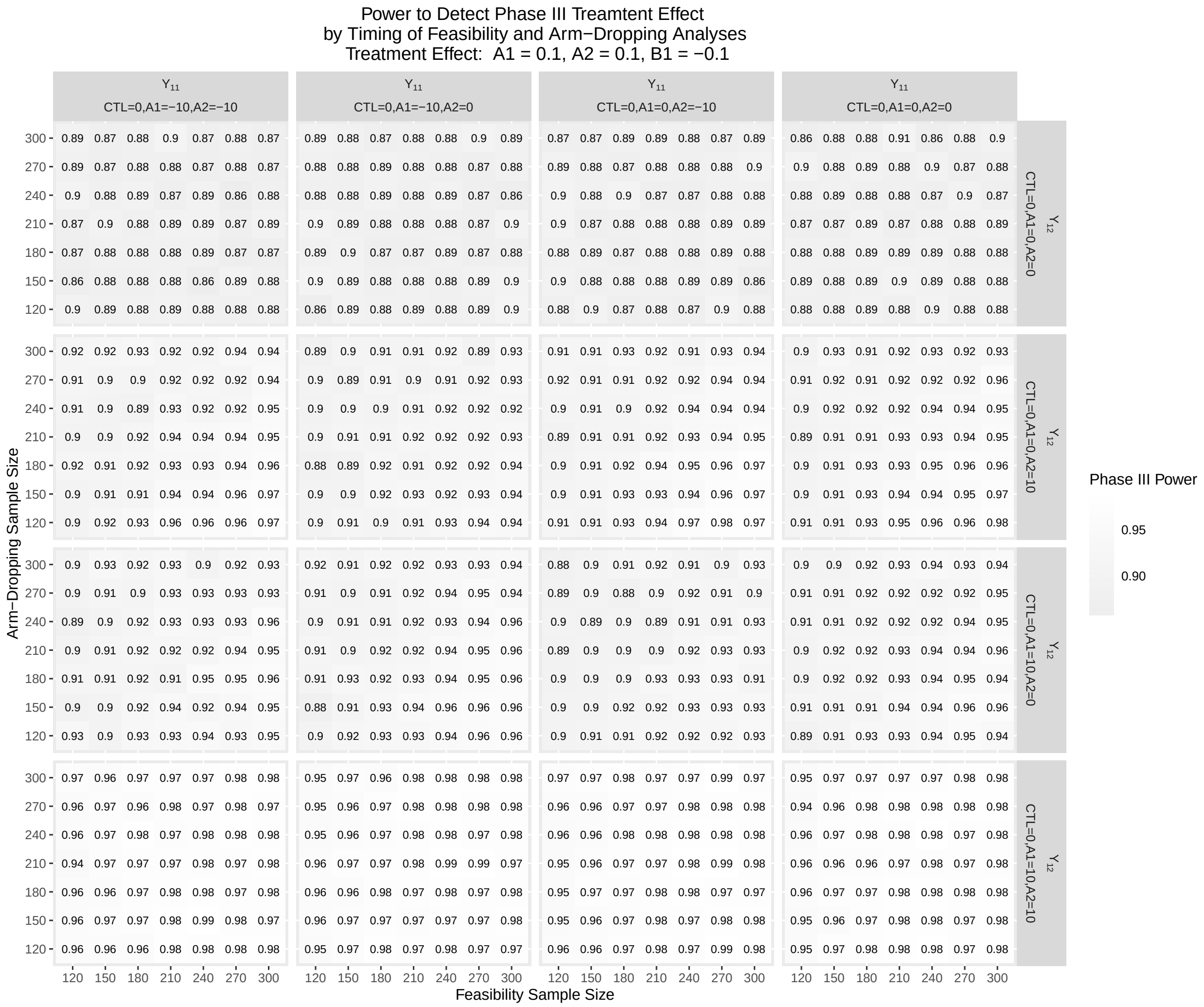
Power to Detect Phase III Treamtent Effect
by Timing of Feasibility and Arm−Dropping Analyses
Treatment Effect: A1 = 0.1, A2 = 0.1, B1 = −0.1
Y11 CTL=0,A1=−10,A2=−10
Y11 CTL=0,A1=−10,A2=0
Y11 CTL=0,A1=0,A2=−10
Y11 CTL=0,A1=0,A2=0
Y12 CTL=0,A1=0,A2=0
Y12 CTL=0,A1=0,A2=10
Y12 CTL=0,A1=10,A2=0
Y12 CTL=0,A1=10,A2=10
Arm−Dropping Sample Size
Feasibility Sample Size
Phase III Power
0.95
0.90

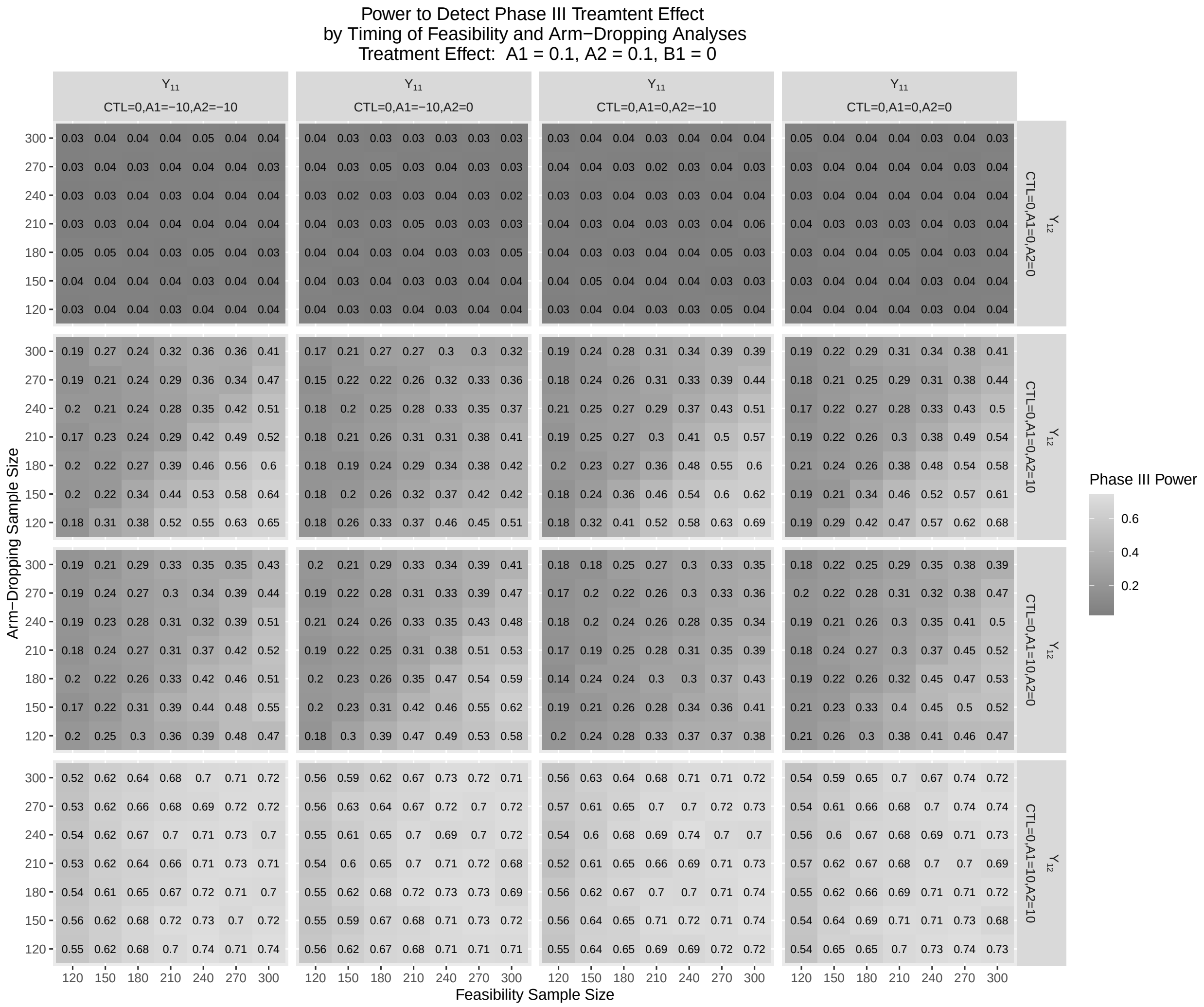
Power to Detect Phase III Treamtent Effect
by Timing of Feasibility and Arm−Dropping Analyses
Treatment Effect: A1 = 0.1, A2 = 0.1, B1 = 0
Y11
CTL=0,A1=−10,A2=−10
CTL=0,A1=−10,A2=0
CTL=0,A1=0,A2=−10
CTL=0,A1=0,A2=0
Y12
CTL=0,A1=0,A2=0
CTL=0,A1=0,A2=10
CTL=0,A1=10,A2=0
CTL=0,A1=10,A2=10
Arm−Dropping Sample Size
Feasibility Sample Size
Phase III Power
0.6
0.4
0.2

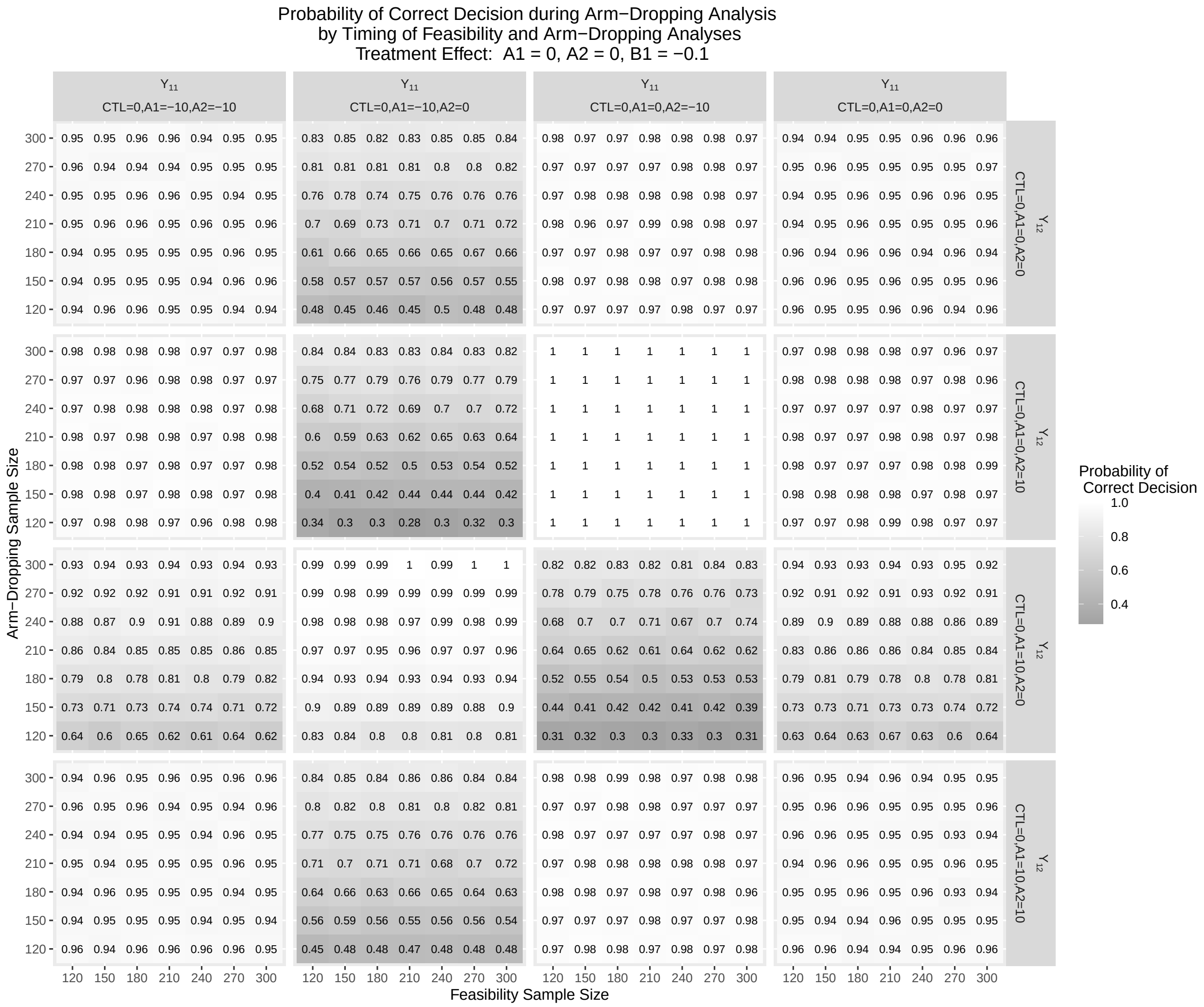
Probability of Correct Decision during Arm−Dropping Analysis
by Timing of Feasibility and Arm−Dropping Analyses
Treatment Effect: A1 = 0, A2 = 0, B1 = −0.1
Y11
CTL=0,A1=−10,A2=−10
CTL=0,A1=−10,A2=0
CTL=0,A1=0,A2=−10
CTL=0,A1=0,A2=0
Y12
CTL=0,A1=0,A2=0
CTL=0,A1=0,A2=10
CTL=0,A1=10,A2=0
CTL=0,A1=10,A2=10
Arm−Dropping Sample Size
Feasibility Sample Size
120 150 180 210 240 270 300
Probability of
Correct Decision
1.0
0.8
0.6
0.4

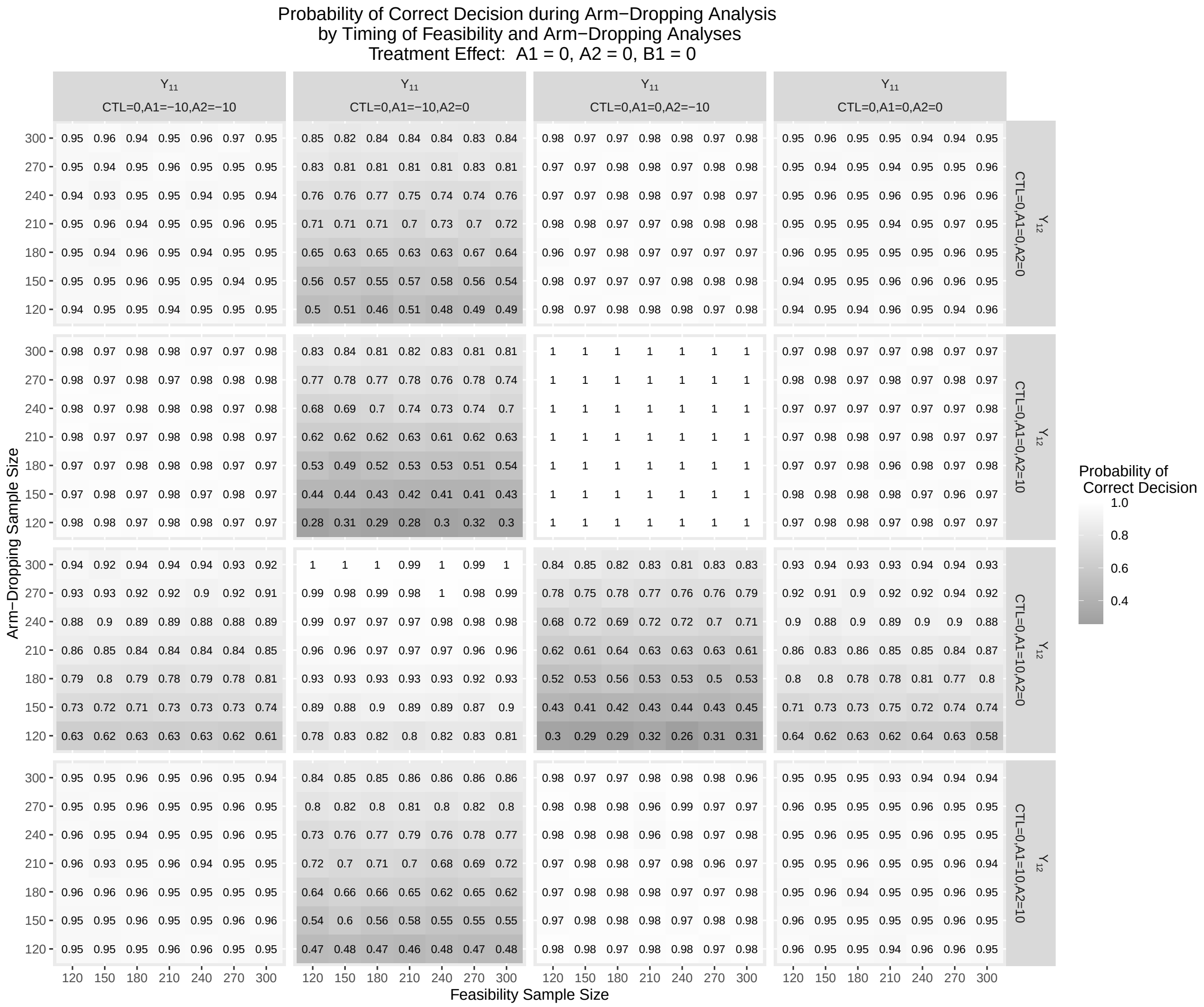
Probability of Correct Decision during Arm−Dropping Analysis
by Timing of Feasibility and Arm−Dropping Analyses
Treatment Effect: A1 = 0, A2 = 0, B1 = 0
Y11 CTL=0,A1=−10,A2=−10
Y11 CTL=0,A1=−10,A2=0
Y11 CTL=0,A1=0,A2=−10
Y11 CTL=0,A1=0,A2=0
Y12 CTL=0,A1=0,A2=0
Y12 CTL=0,A1=0,A2=10
Y12 CTL=0,A1=10,A2=0
Y12 CTL=0,A1=10,A2=10
Arm−Dropping Sample Size
Feasibility Sample Size
Probability of Correct Decision
1.0
0.8
0.6
0.4

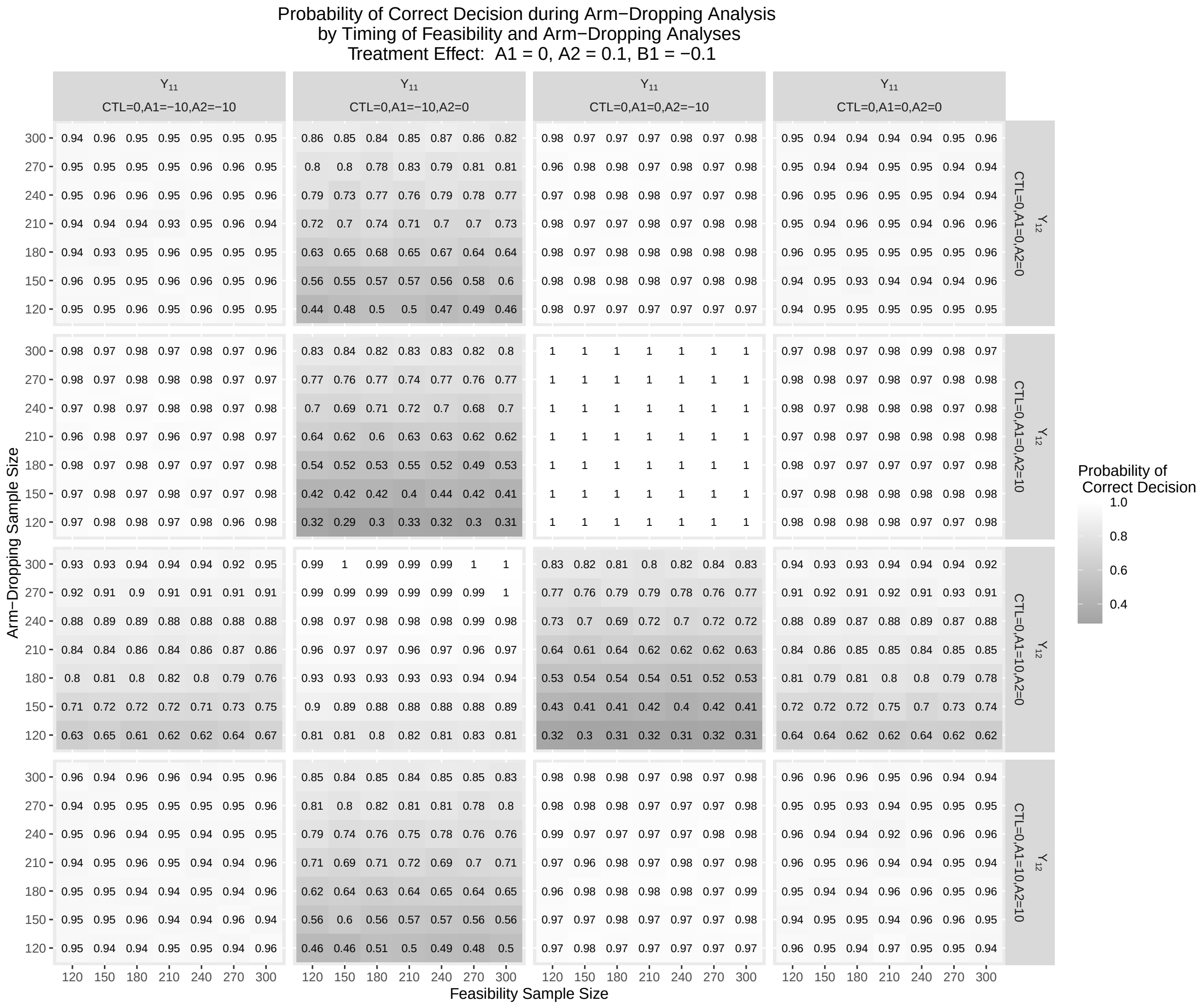
Probability of Correct Decision during Arm−Dropping Analysis
by Timing of Feasibility and Arm−Dropping Analyses
Treatment Effect: A1 = 0, A2 = 0.1, B1 = −0.1
Y_{11} CTL=0,A1=−10,A2=−10
Y_{11} CTL=0,A1=−10,A2=0
Y_{11} CTL=0,A1=0,A2=−10
Y_{11} CTL=0,A1=0,A2=0
Y_{12} CTL=0,A1=0,A2=0
Y_{12} CTL=0,A1=0,A2=10
Y_{12} CTL=0,A1=10,A2=0
Y_{12} CTL=0,A1=10,A2=10
Arm−Dropping Sample Size
Feasibility Sample Size
120 150 180 210 240 270 300
Probability of Correct Decision
1.0
0.8
0.6
0.4

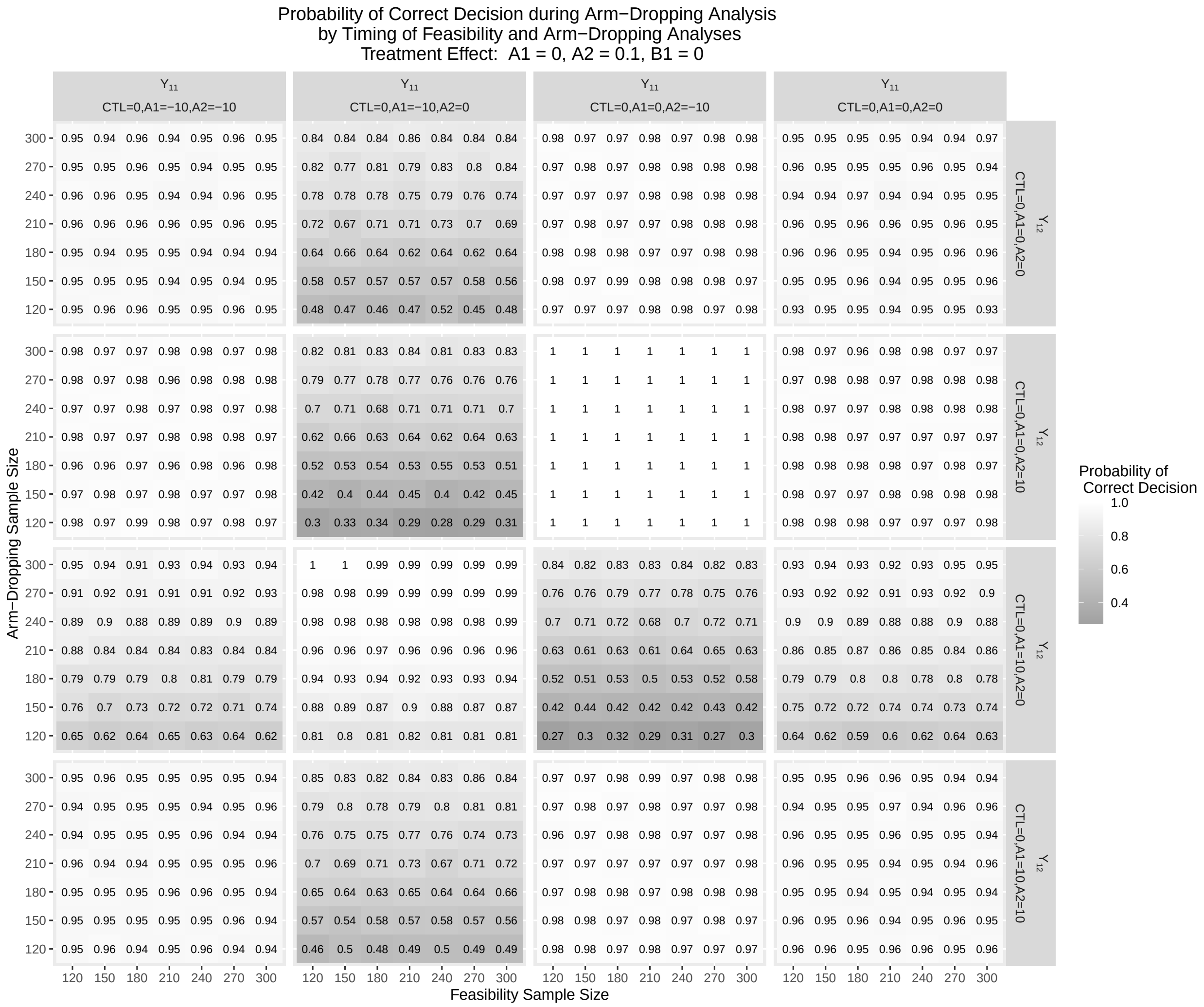
Probability of Correct Decision during Arm−Dropping Analysis
by Timing of Feasibility and Arm−Dropping Analyses
Treatment Effect: A1 = 0, A2 = 0.1, B1 = 0
Y11 CTL=0,A1=−10,A2=−10
Y11 CTL=0,A1=−10,A2=0
Y11 CTL=0,A1=0,A2=−10
Y11 CTL=0,A1=0,A2=0
Y12 CTL=0,A1=0,A2=0
Y12 CTL=0,A1=0,A2=10
Y12 CTL=0,A1=10,A2=0
Y12 CTL=0,A1=10,A2=10
Arm−Dropping Sample Size
Feasibility Sample Size
120 150 180 210 240 270 300
Probability of Correct Decision
1.0
0.8
0.6
0.4

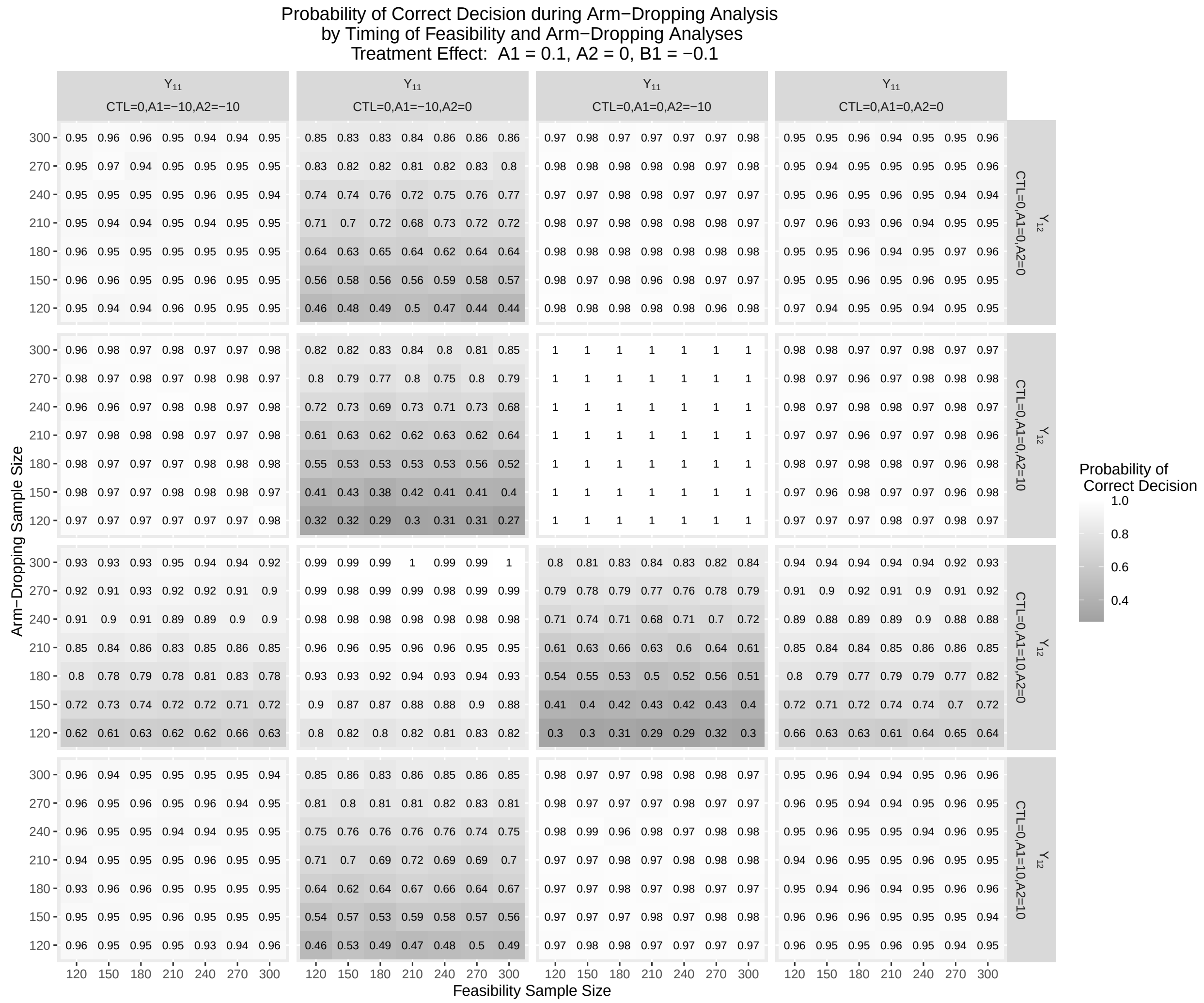
Probability of Correct Decision during Arm−Dropping Analysis
by Timing of Feasibility and Arm−Dropping Analyses
Treatment Effect: A1 = 0.1, A2 = 0, B1 = −0.1
Y11
CTL=0,A1=−10,A2=−10
CTL=0,A1=−10,A2=0
CTL=0,A1=0,A2=−10
CTL=0,A1=0,A2=0
Y12
CTL=0,A1=0,A2=0
CTL=0,A1=0,A2=10
CTL=0,A1=10,A2=0
CTL=0,A1=10,A2=10
Arm−Dropping Sample Size
Feasibility Sample Size
120 150 180 210 240 270 300
Probability of
Correct Decision
1.0
0.8
0.6
0.4

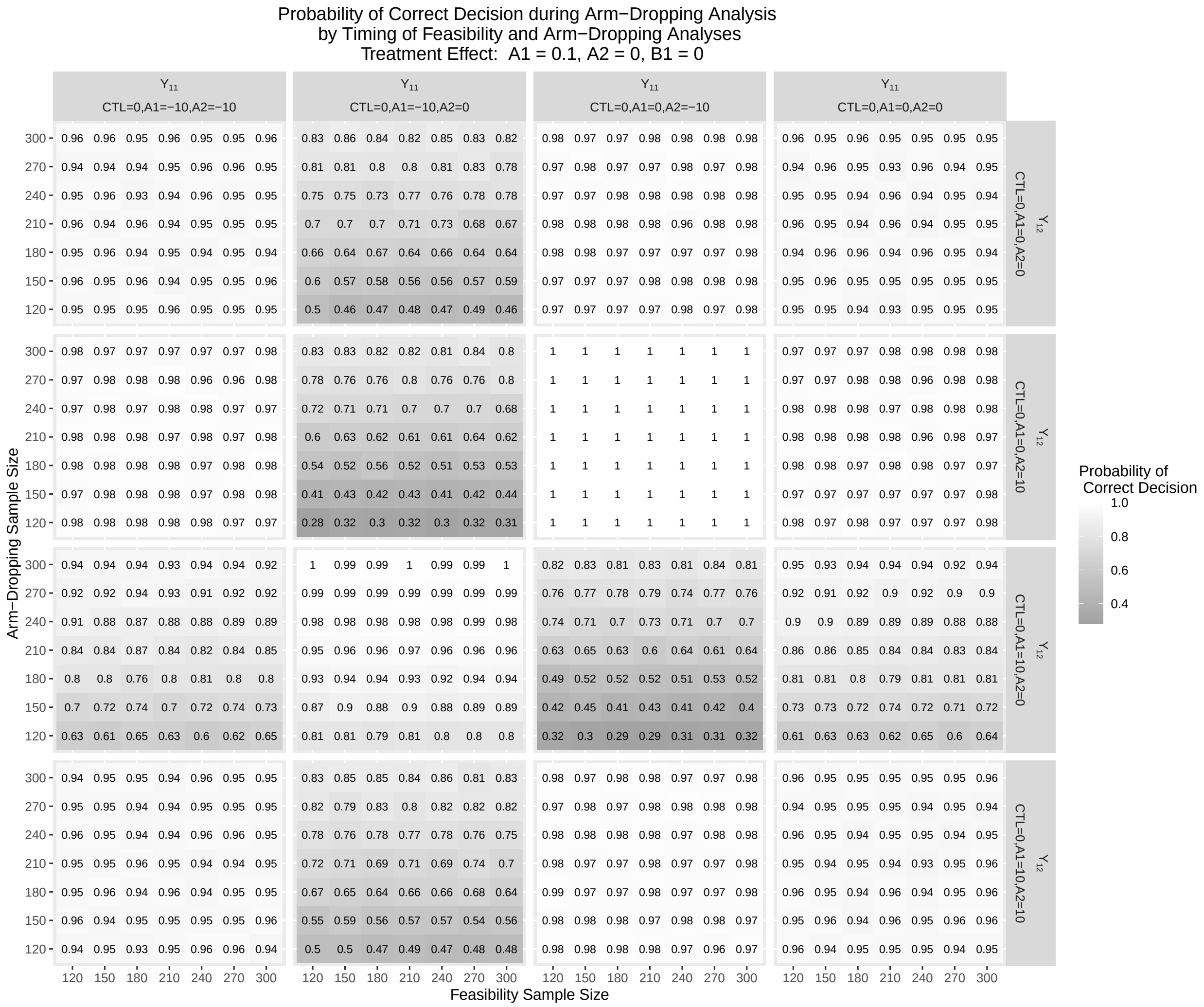
Probability of Correct Decision during Arm−Dropping Analysis
by Timing of Feasibility and Arm−Dropping Analyses
Treatment Effect: A1 = 0.1, A2 = 0, B1 = 0
Y11
CTL=0,A1=−10,A2=−10
CTL=0,A1=−10,A2=0
CTL=0,A1=0,A2=−10
CTL=0,A1=0,A2=0
Y12
CTL=0,A1=0,A2=0
CTL=0,A1=0,A2=10
CTL=0,A1=10,A2=0
CTL=0,A1=10,A2=10
Arm−Dropping Sample Size
Feasibility Sample Size
Probability of
Correct Decision
1.0
0.8
0.6
0.4

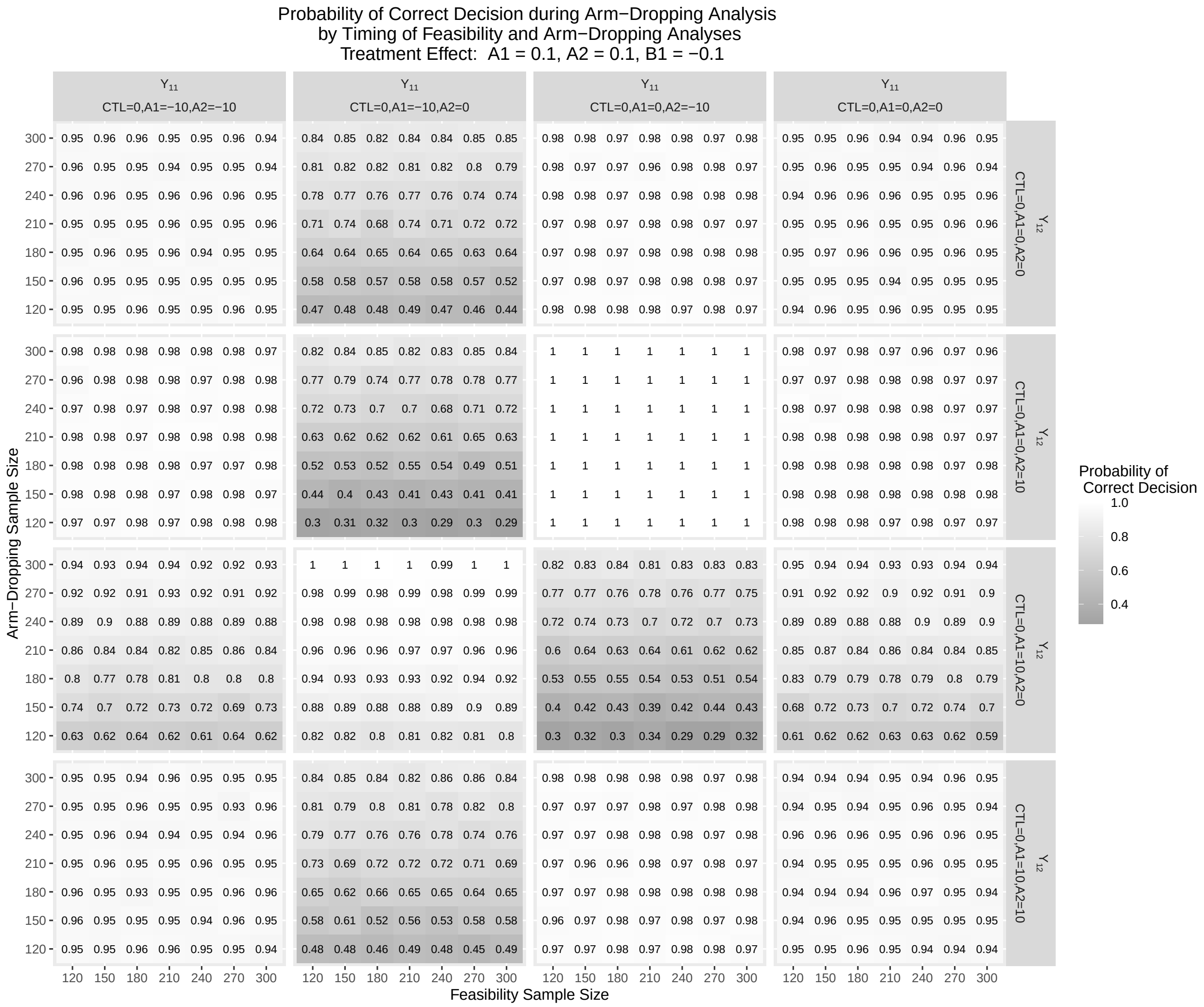
Probability of Correct Decision during Arm−Dropping Analysis
by Timing of Feasibility and Arm−Dropping Analyses
Treatment Effect: A1 = 0.1, A2 = 0.1, B1 = −0.1
Y11
CTL=0,A1=−10,A2=−10
CTL=0,A1=−10,A2=0
CTL=0,A1=0,A2=−10
CTL=0,A1=0,A2=0
Y12
CTL=0,A1=0,A2=0
CTL=0,A1=0,A2=10
CTL=0,A1=10,A2=0
CTL=0,A1=10,A2=10
Arm−Dropping Sample Size
Feasibility Sample Size
Probability of Correct Decision

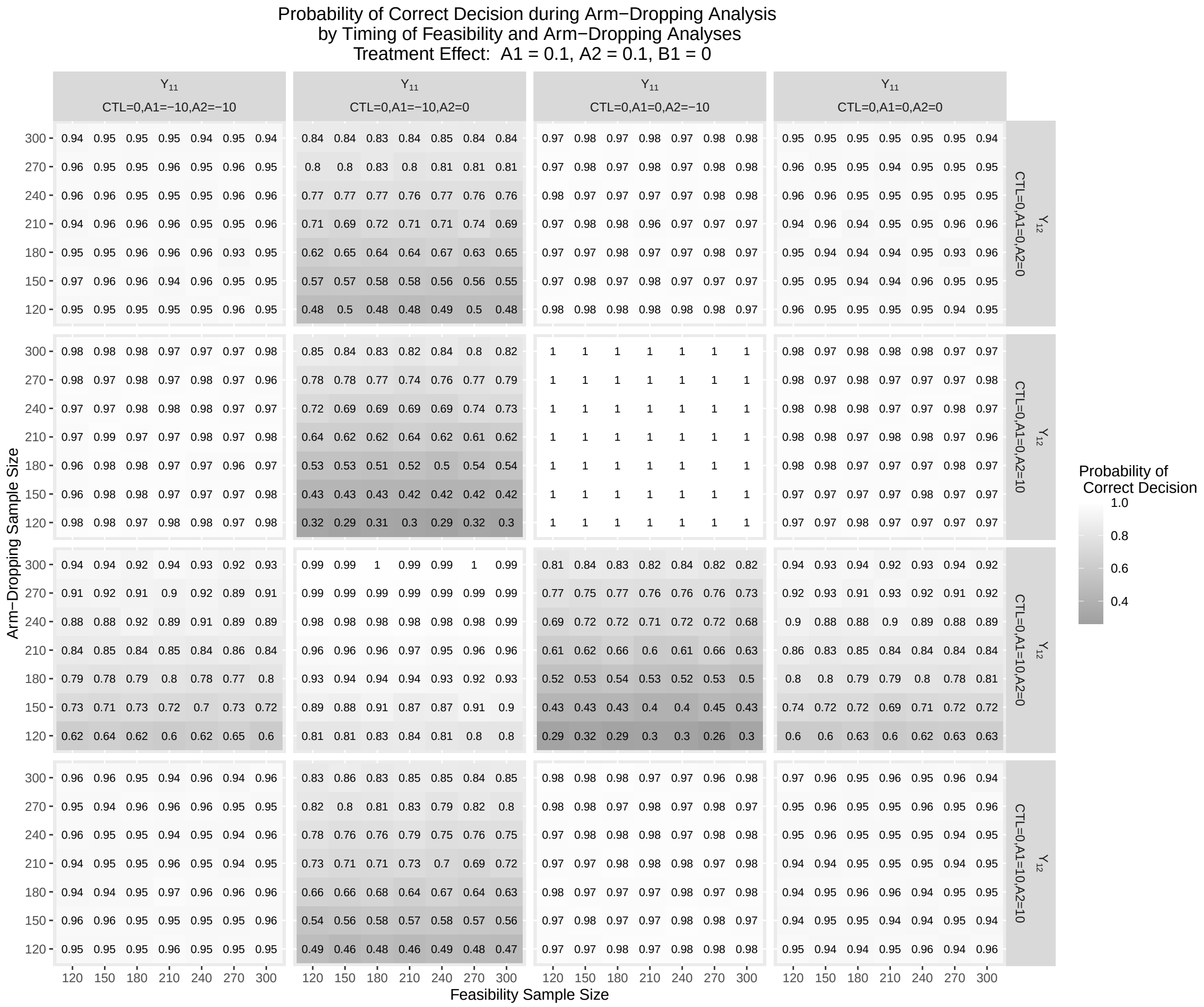
Probability of Correct Decision during Arm−Dropping Analysis
by Timing of Feasibility and Arm−Dropping Analyses
Treatment Effect: A1 = 0.1, A2 = 0.1, B1 = 0
Y11 CTL=0,A1=−10,A2=−10
Y11 CTL=0,A1=−10,A2=0
Y11 CTL=0,A1=0,A2=−10
Y11 CTL=0,A1=0,A2=0
Y12 CTL=0,A1=0,A2=0
300 0.94 0.95 0.95 0.95 0.94 0.95 0.94 0.84 0.84 0.83 0.84 0.85 0.84 0.84 0.97 0.98 0.97 0.98 0.97 0.98 0.98 0.95 0.95 0.95 0.95 0.95 0.95 0.94
270 0.96 0.95 0.95 0.96 0.95 0.96 0.95 0.8 0.8 0.83 0.8 0.81 0.81 0.81 0.97 0.98 0.97 0.98 0.97 0.98 0.98 0.96 0.95 0.95 0.94 0.95 0.95 0.95
240 0.96 0.96 0.95 0.95 0.95 0.96 0.96 0.77 0.77 0.77 0.76 0.77 0.76 0.76 0.98 0.97 0.97 0.97 0.97 0.98 0.98 0.96 0.96 0.95 0.95 0.95 0.95 0.95
210 0.94 0.96 0.96 0.96 0.95 0.95 0.96 0.71 0.69 0.72 0.71 0.71 0.74 0.69 0.97 0.98 0.98 0.96 0.97 0.97 0.97 0.94 0.96 0.94 0.95 0.95 0.96 0.96
180 0.95 0.95 0.96 0.96 0.96 0.93 0.95 0.62 0.65 0.64 0.64 0.67 0.63 0.65 0.97 0.97 0.98 0.97 0.97 0.98 0.97 0.95 0.94 0.94 0.94 0.95 0.93 0.96
150 0.97 0.96 0.96 0.94 0.96 0.95 0.95 0.57 0.57 0.58 0.58 0.56 0.56 0.55 0.97 0.98 0.97 0.98 0.97 0.97 0.97 0.95 0.95 0.94 0.94 0.96 0.95 0.95
120 0.95 0.95 0.95 0.95 0.95 0.96 0.95 0.48 0.5 0.48 0.48 0.49 0.5 0.48 0.98 0.98 0.98 0.98 0.98 0.98 0.97 0.96 0.95 0.95 0.95 0.95 0.94 0.95
Y12 CTL=0,A1=0,A2=10
300 0.98 0.98 0.98 0.97 0.97 0.97 0.98 0.85 0.84 0.83 0.82 0.84 0.8 0.82 1 1 1 1 1 1 1 0.98 0.97 0.98 0.98 0.98 0.97 0.97
270 0.98 0.97 0.98 0.97 0.98 0.97 0.96 0.78 0.78 0.77 0.74 0.76 0.77 0.79 1 1 1 1 1 1 1 0.98 0.97 0.98 0.97 0.97 0.97 0.98
240 0.97 0.97 0.98 0.98 0.98 0.97 0.97 0.72 0.69 0.69 0.69 0.69 0.74 0.73 1 1 1 1 1 1 1 0.98 0.98 0.98 0.97 0.97 0.98 0.97
210 0.97 0.99 0.97 0.97 0.98 0.97 0.98 0.64 0.62 0.62 0.64 0.62 0.61 0.62 1 1 1 1 1 1 1 0.98 0.98 0.97 0.98 0.98 0.97 0.96
180 0.96 0.98 0.98 0.97 0.97 0.96 0.97 0.53 0.53 0.51 0.52 0.5 0.54 0.54 1 1 1 1 1 1 1 0.98 0.98 0.97 0.97 0.97 0.98 0.97
150 0.96 0.98 0.98 0.97 0.97 0.97 0.98 0.43 0.43 0.43 0.42 0.42 0.42 0.42 1 1 1 1 1 1 1 0.97 0.97 0.97 0.97 0.98 0.97 0.97
120 0.98 0.98 0.97 0.98 0.98 0.97 0.98 0.32 0.29 0.31 0.3 0.29 0.32 0.3 1 1 1 1 1 1 1 0.97 0.97 0.98 0.97 0.97 0.97 0.97
Y12 CTL=0,A1=10,A2=0
300 0.94 0.94 0.92 0.94 0.93 0.92 0.93 0.99 0.99 1 0.99 0.99 1 0.99 0.81 0.84 0.83 0.82 0.84 0.82 0.82 0.94 0.93 0.94 0.92 0.93 0.94 0.92
270 0.91 0.92 0.91 0.9 0.92 0.89 0.91 0.99 0.99 0.99 0.99 0.99 0.99 0.99 0.77 0.75 0.77 0.76 0.76 0.76 0.73 0.92 0.93 0.91 0.93 0.92 0.91 0.92
240 0.88 0.88 0.92 0.89 0.91 0.89 0.89 0.98 0.98 0.98 0.98 0.98 0.98 0.99 0.69 0.72 0.72 0.71 0.72 0.72 0.68 0.9 0.88 0.88 0.9 0.89 0.88 0.89
210 0.84 0.85 0.84 0.85 0.84 0.86 0.84 0.96 0.96 0.96 0.97 0.95 0.96 0.96 0.61 0.62 0.66 0.6 0.61 0.66 0.63 0.86 0.83 0.85 0.84 0.84 0.84 0.84
180 0.79 0.78 0.79 0.8 0.78 0.77 0.8 0.93 0.94 0.94 0.94 0.93 0.92 0.93 0.52 0.53 0.54 0.53 0.52 0.53 0.5 0.8 0.8 0.79 0.79 0.8 0.78 0.81
150 0.73 0.71 0.73 0.72 0.7 0.73 0.72 0.89 0.88 0.91 0.87 0.87 0.91 0.9 0.43 0.43 0.43 0.4 0.4 0.45 0.43 0.74 0.72 0.72 0.69 0.71 0.72 0.72
120 0.62 0.64 0.62 0.6 0.62 0.65 0.6 0.81 0.81 0.83 0.84 0.81 0.8 0.8 0.29 0.32 0.29 0.3 0.3 0.26 0.3 0.6 0.6 0.63 0.6 0.62 0.63 0.63
Y12 CTL=0,A1=10,A2=10
300 0.96 0.96 0.95 0.94 0.96 0.94 0.96 0.83 0.86 0.83 0.85 0.85 0.84 0.85 0.98 0.98 0.98 0.97 0.97 0.96 0.98 0.97 0.96 0.95 0.96 0.95 0.96 0.94
270 0.95 0.94 0.96 0.96 0.96 0.95 0.95 0.82 0.8 0.81 0.83 0.79 0.82 0.8 0.98 0.98 0.97 0.98 0.97 0.98 0.97 0.95 0.96 0.95 0.95 0.96 0.95 0.96
240 0.96 0.95 0.95 0.94 0.95 0.94 0.96 0.78 0.76 0.76 0.79 0.75 0.76 0.75 0.97 0.98 0.98 0.98 0.97 0.98 0.98 0.95 0.96 0.95 0.95 0.95 0.94 0.95
210 0.94 0.95 0.95 0.96 0.95 0.94 0.95 0.73 0.71 0.71 0.73 0.7 0.69 0.72 0.97 0.97 0.98 0.98 0.98 0.97 0.98 0.94 0.94 0.95 0.95 0.95 0.94 0.95
180 0.94 0.94 0.95 0.97 0.96 0.96 0.96 0.66 0.66 0.68 0.64 0.67 0.64 0.63 0.98 0.97 0.97 0.97 0.98 0.97 0.98 0.94 0.95 0.96 0.96 0.94 0.95 0.95
150 0.96 0.96 0.95 0.95 0.95 0.95 0.96 0.54 0.56 0.58 0.57 0.58 0.57 0.56 0.97 0.98 0.97 0.97 0.98 0.98 0.97 0.94 0.95 0.95 0.94 0.94 0.95 0.94
120 0.95 0.95 0.95 0.96 0.95 0.95 0.95 0.49 0.46 0.48 0.46 0.49 0.48 0.47 0.97 0.97 0.98 0.97 0.98 0.98 0.98 0.95 0.94 0.94 0.95 0.96 0.94 0.96
120 150 180 210 240 270 300 120 150 180 210 240 270 300 120 150 180 210 240 270 300 120 150 180 210 240 270 300
Feasibility Sample Size
Arm−Dropping Sample Size
Probability of Correct Decision
1.0
0.8
0.6
0.4

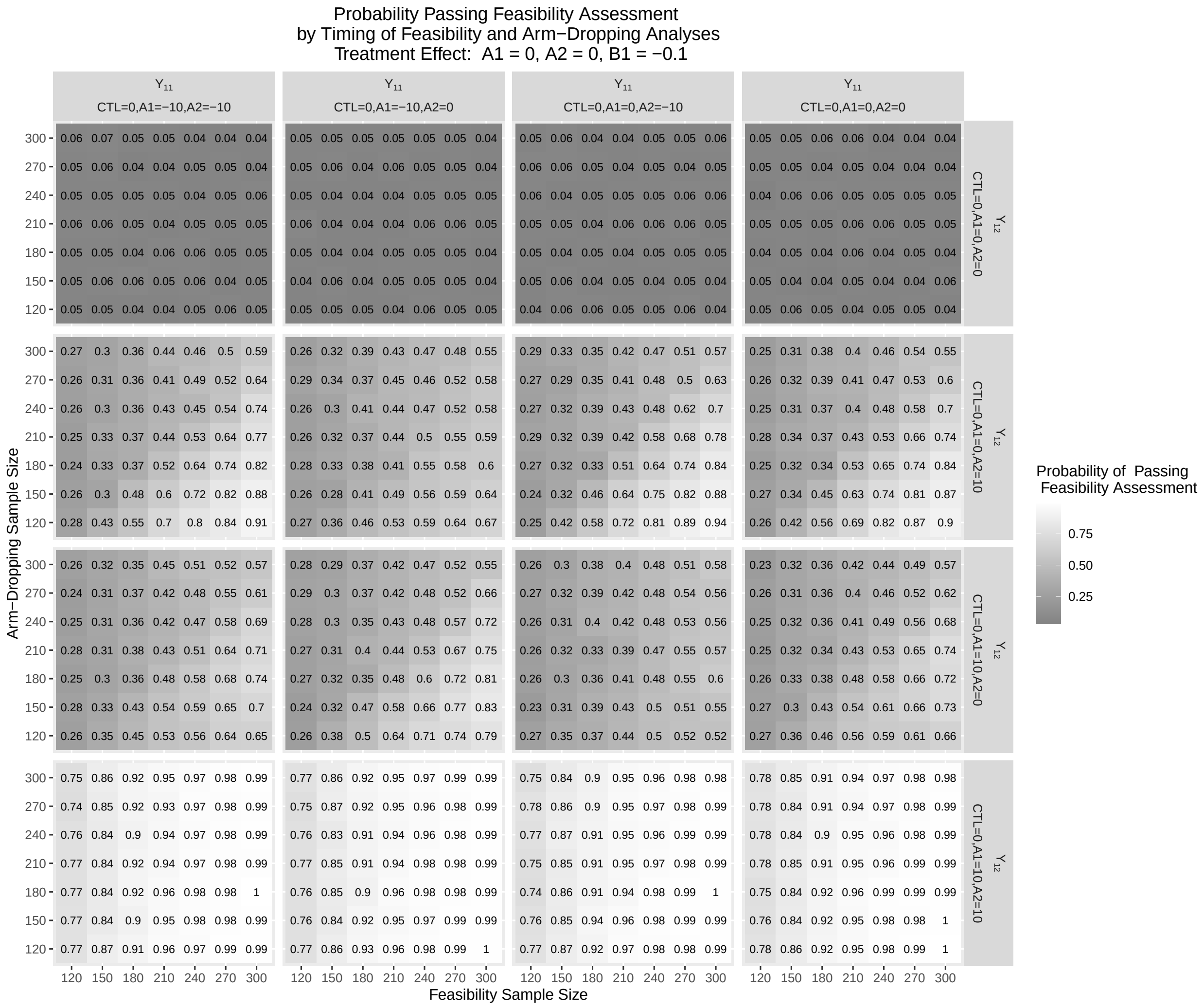
Probability Passing Feasibility Assessment
by Timing of Feasibility and Arm−Dropping Analyses
Treatment Effect: A1 = 0, A2 = 0, B1 = −0.1
Y11
CTL=0,A1=−10,A2=−10
CTL=0,A1=−10,A2=0
CTL=0,A1=0,A2=−10
CTL=0,A1=0,A2=0
Y12
CTL=0,A1=0,A2=0
CTL=0,A1=0,A2=10
CTL=0,A1=10,A2=0
CTL=0,A1=10,A2=10
Arm−Dropping Sample Size
Feasibility Sample Size
Probability of Passing
Feasibility Assessment
0.75
0.50
0.25

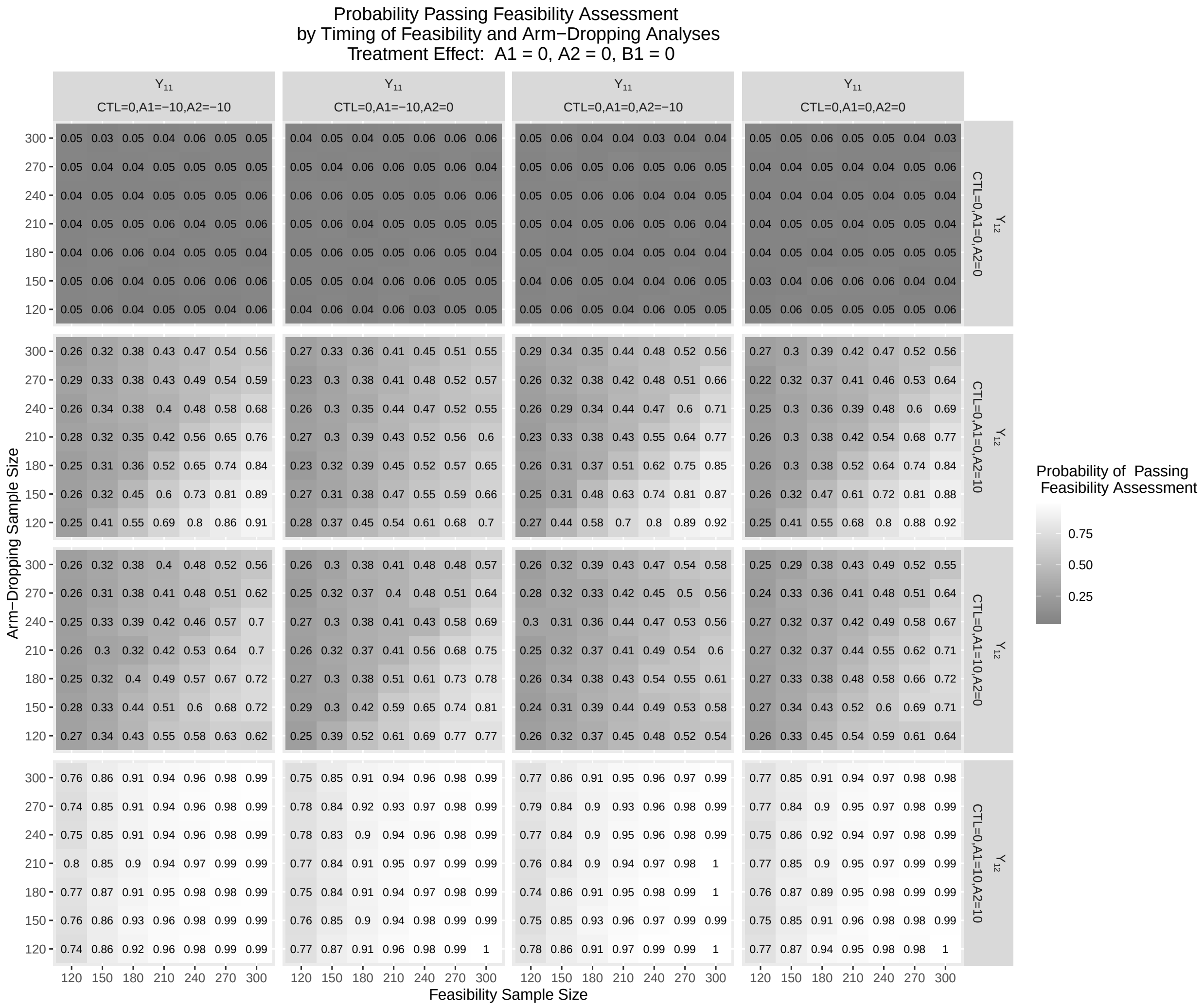

Probability Passing Feasibility Assessment
by Timing of Feasibility and Arm−Dropping Analyses
Treatment Effect: A1 = 0, A2 = 0, B1 = 0
Y_{11}
CTL=0,A1=−10,A2=−10
CTL=0,A1=−10,A2=0
CTL=0,A1=0,A2=−10
CTL=0,A1=0,A2=0
Y_{12}
CTL=0,A1=0,A2=0
CTL=0,A1=0,A2=10
CTL=0,A1=10,A2=0
CTL=0,A1=10,A2=10
Arm−Dropping Sample Size
Feasibility Sample Size
120 150 180 210 240 270 300
Probability of Passing
Feasibility Assessment
0.75
0.50
0.25

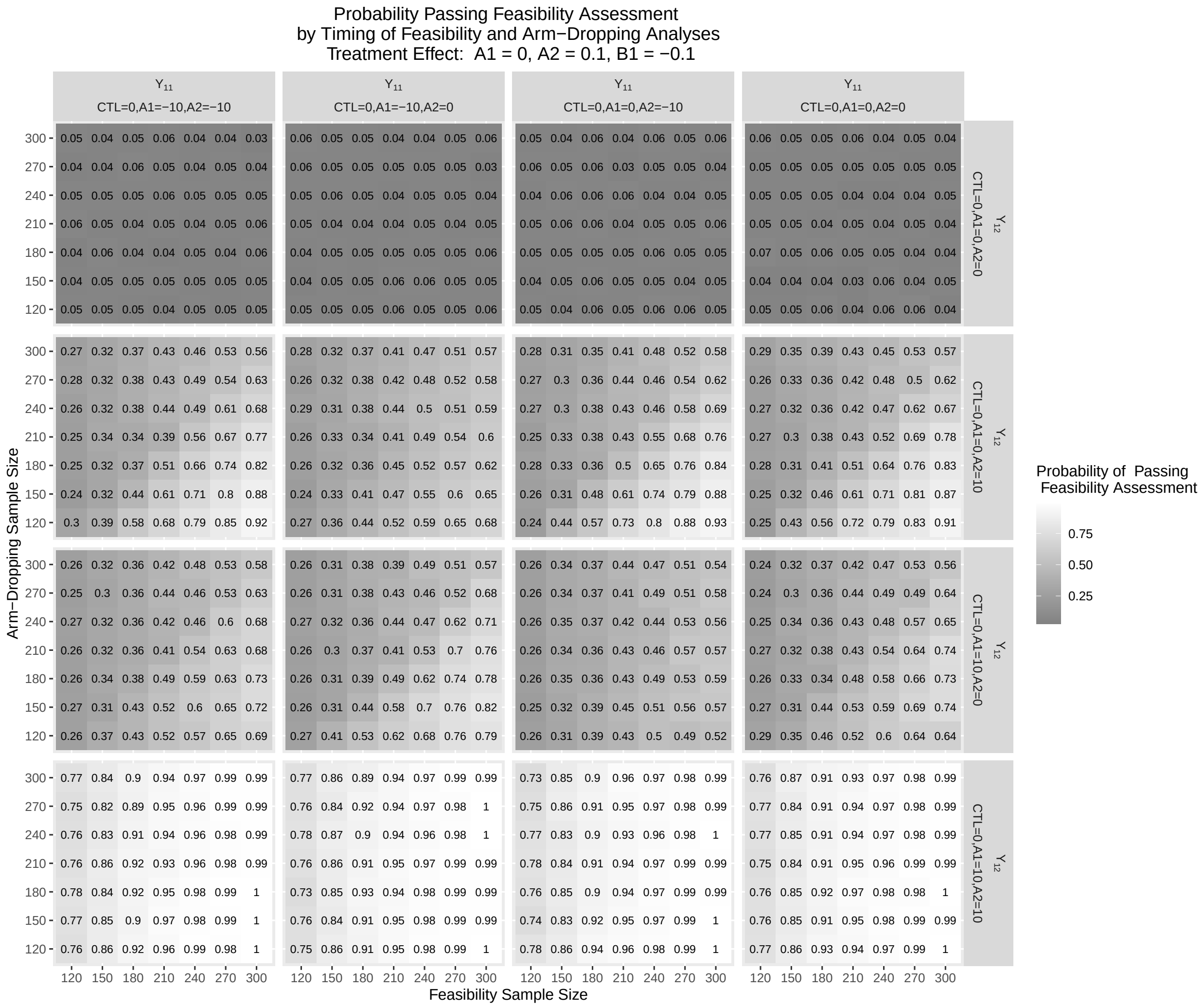

Probability Passing Feasibility Assessment
by Timing of Feasibility and Arm−Dropping Analyses
Treatment Effect: A1 = 0, A2 = 0.1, B1 = −0.1
Y11
CTL=0,A1=−10,A2=−10
CTL=0,A1=−10,A2=0
CTL=0,A1=0,A2=−10
CTL=0,A1=0,A2=0
Y12
CTL=0,A1=0,A2=0
CTL=0,A1=0,A2=10
CTL=0,A1=10,A2=0
CTL=0,A1=10,A2=10
Arm−Dropping Sample Size
Feasibility Sample Size
Probability of Passing
Feasibility Assessment
0.75
0.50
0.25

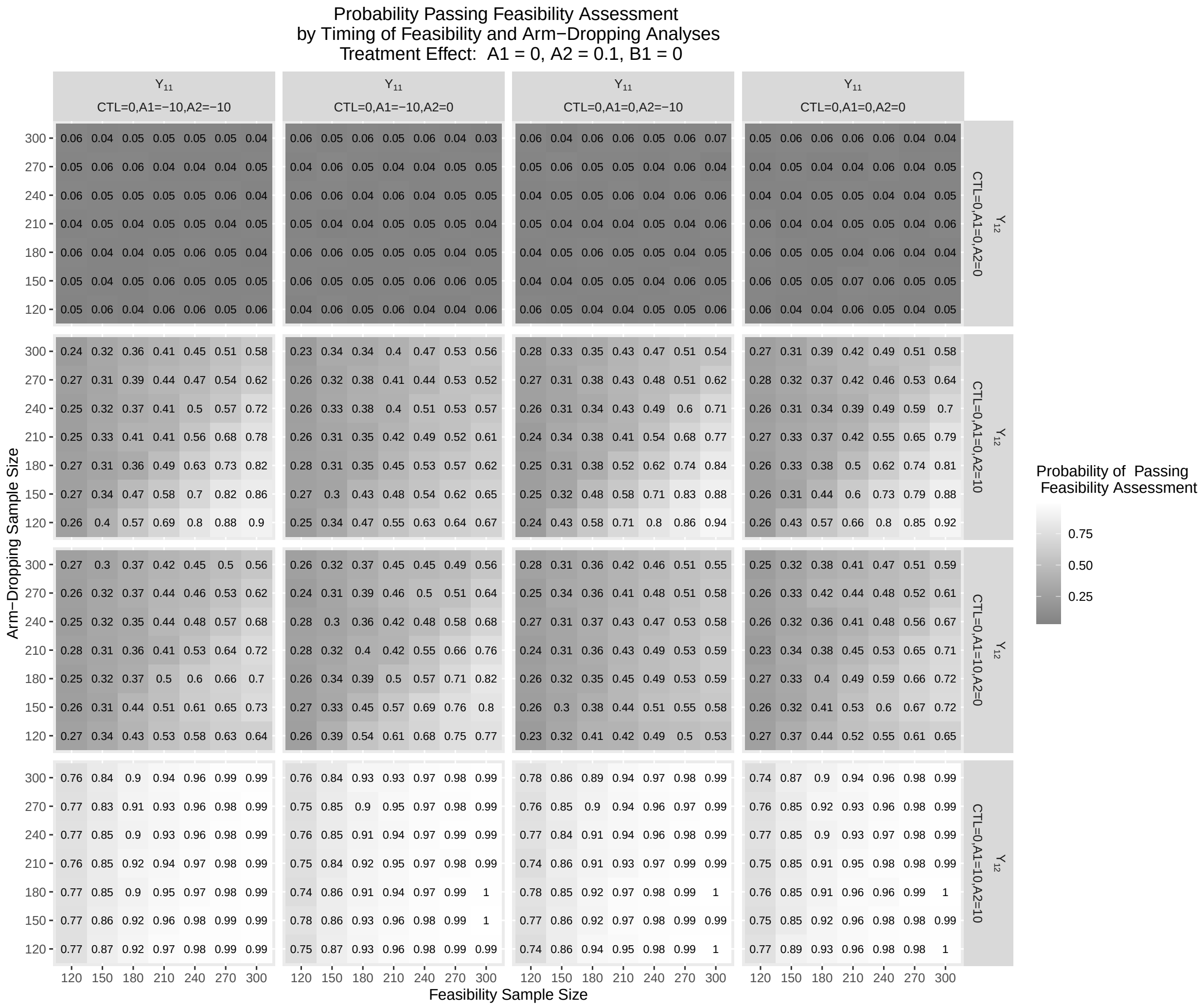

Probability Passing Feasibility Assessment
by Timing of Feasibility and Arm−Dropping Analyses
Treatment Effect: A1 = 0, A2 = 0.1, B1 = 0
Y_{11} CTL=0,A1=−10,A2=−10
Y_{11} CTL=0,A1=−10,A2=0
Y_{11} CTL=0,A1=0,A2=−10
Y_{11} CTL=0,A1=0,A2=0
Y_{12} CTL=0,A1=0,A2=0
Y_{12} CTL=0,A1=0,A2=10
Y_{12} CTL=0,A1=10,A2=0
Y_{12} CTL=0,A1=10,A2=10
Arm−Dropping Sample Size
Feasibility Sample Size
120 150 180 210 240 270 300
Probability of Passing
Feasibility Assessment
0.75
0.50
0.25

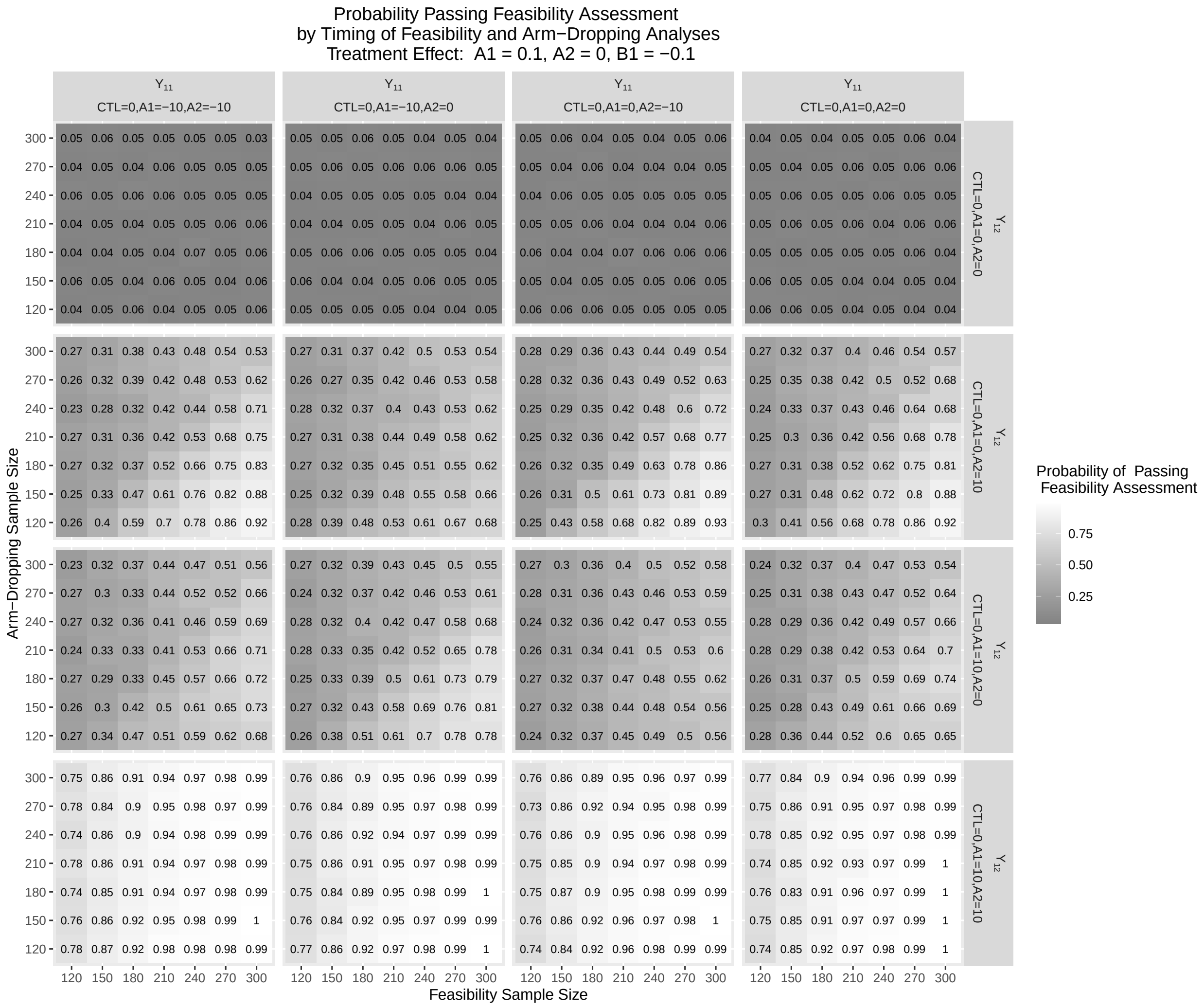
Probability Passing Feasibility Assessment
by Timing of Feasibility and Arm−Dropping Analyses
Treatment Effect: A1 = 0.1, A2 = 0, B1 = −0.1
Y11
CTL=0,A1=−10,A2=−10
CTL=0,A1=−10,A2=0
CTL=0,A1=0,A2=−10
CTL=0,A1=0,A2=0
Y12
CTL=0,A1=0,A2=0
CTL=0,A1=0,A2=10
CTL=0,A1=10,A2=0
CTL=0,A1=10,A2=10
Arm−Dropping Sample Size
Feasibility Sample Size
120 150 180 210 240 270 300
Probability of Passing
Feasibility Assessment
0.75
0.50
0.25

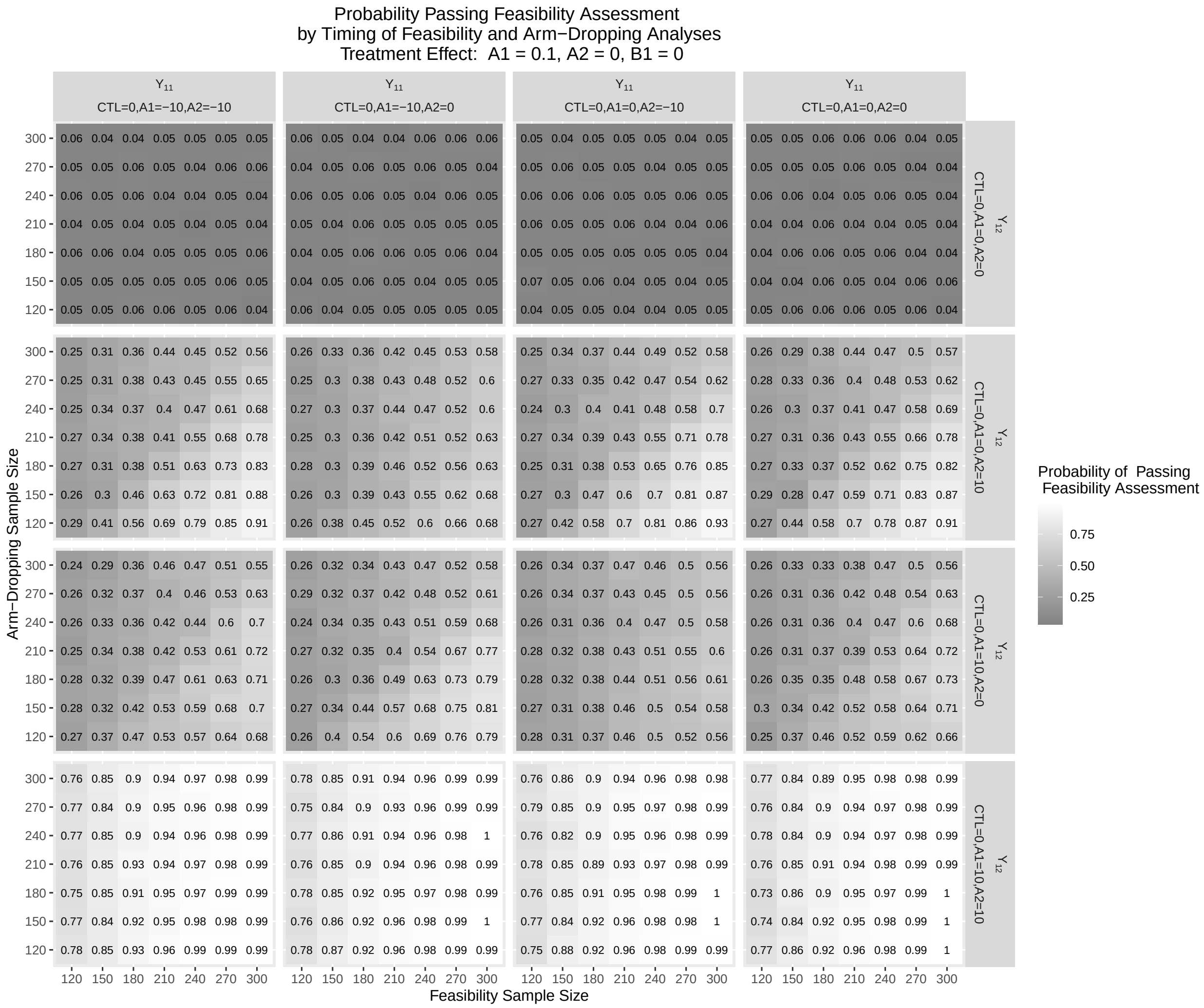
Probability Passing Feasibility Assessment
by Timing of Feasibility and Arm−Dropping Analyses
Treatment Effect: A1 = 0.1, A2 = 0, B1 = 0
Y11
CTL=0,A1=−10,A2=−10
CTL=0,A1=−10,A2=0
CTL=0,A1=0,A2=−10
CTL=0,A1=0,A2=0
Y12
CTL=0,A1=0,A2=0
CTL=0,A1=0,A2=10
CTL=0,A1=10,A2=0
CTL=0,A1=10,A2=10
Arm−Dropping Sample Size
Feasibility Sample Size
120 150 180 210 240 270 300
Probability of Passing
Feasibility Assessment
0.75
0.50
0.25

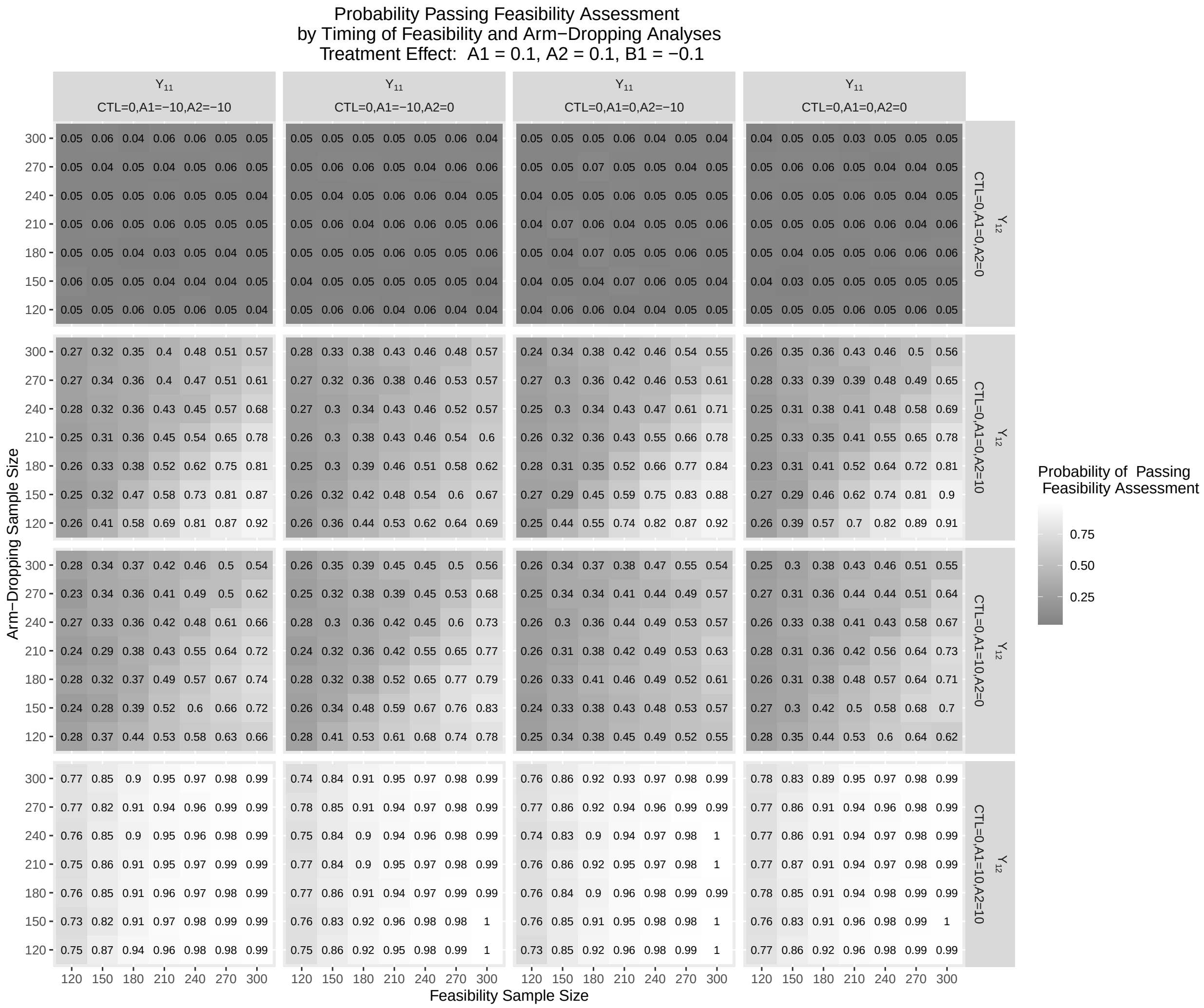
Probability Passing Feasibility Assessment
by Timing of Feasibility and Arm−Dropping Analyses
Treatment Effect: A1 = 0.1, A2 = 0.1, B1 = −0.1
Y_{11} CTL=0,A1=−10,A2=−10
Y_{11} CTL=0,A1=−10,A2=0
Y_{11} CTL=0,A1=0,A2=−10
Y_{11} CTL=0,A1=0,A2=0
Y_{12} CTL=0,A1=0,A2=0
Y_{12} CTL=0,A1=0,A2=10
Y_{12} CTL=0,A1=10,A2=0
Y_{12} CTL=0,A1=10,A2=10
Arm−Dropping Sample Size
Feasibility Sample Size
120 150 180 210 240 270 300
Probability of Passing Feasibility Assessment
0.75
0.50
0.25

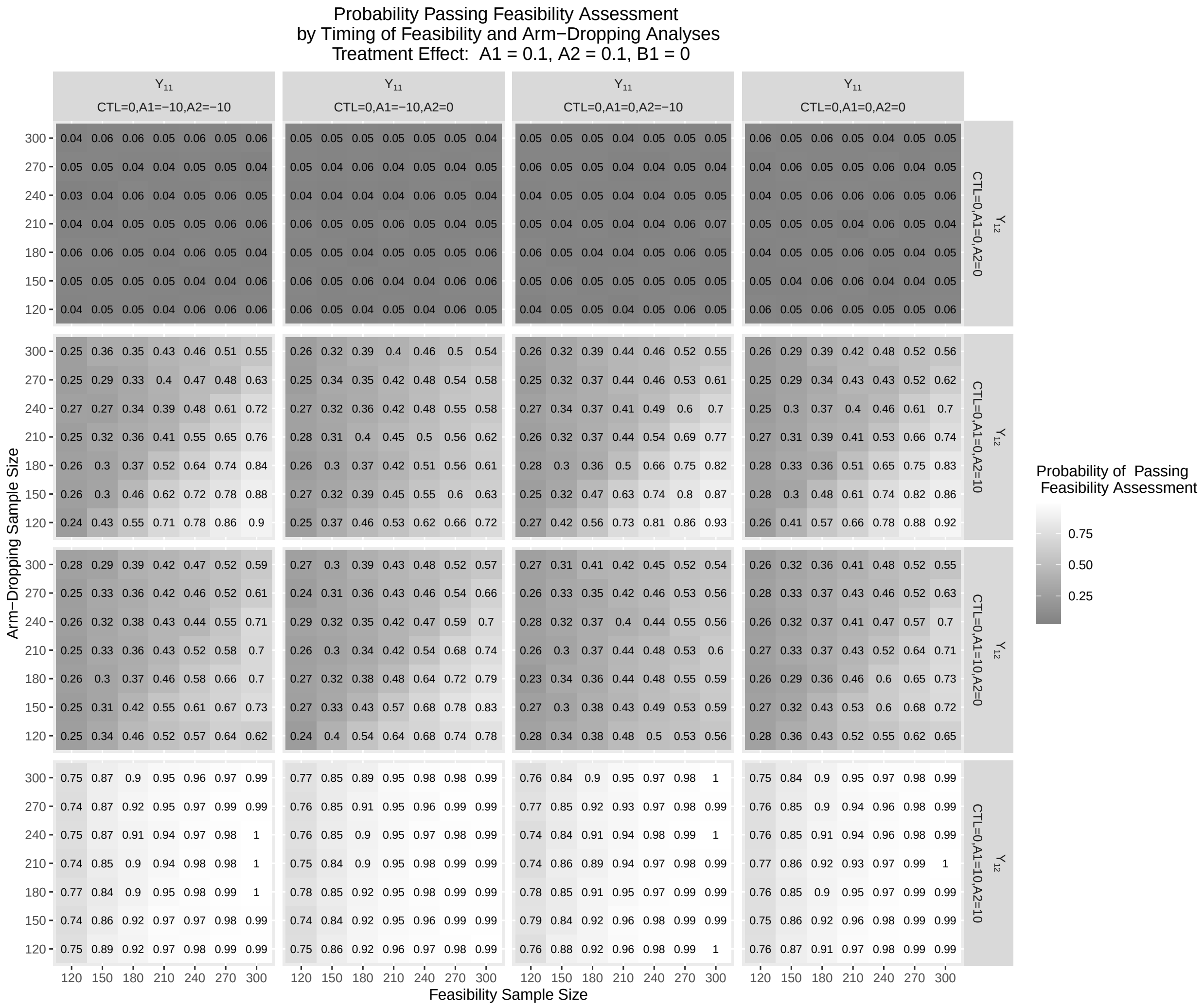
Probability Passing Feasibility Assessment
by Timing of Feasibility and Arm−Dropping Analyses
Treatment Effect: A1 = 0.1, A2 = 0.1, B1 = 0
Y_{11}
CTL=0,A1=−10,A2=−10
CTL=0,A1=−10,A2=0
CTL=0,A1=0,A2=−10
CTL=0,A1=0,A2=0
Y_{12}
CTL=0,A1=0,A2=0
CTL=0,A1=0,A2=10
CTL=0,A1=10,A2=0
CTL=0,A1=10,A2=10
Arm−Dropping Sample Size
Feasibility Sample Size
Probability of Passing
Feasibility Assessment
0.75
0.50
0.25
