# Supplementary material for: Design considerations for Factorial Adaptive Multi-Arm Multi-Stage (FAST) clinical trials
Source: Trials. 2024 Sep 12;25:608. doi: 10.1186/s13063-024-08400-6 (PMC11391813; doi:10.1186/s13063-024-08400-6)
Supplement: Supplementary file 2 — Supplementary Material 2. [file 13063_2024_8400_MOESM2_ESM.pdf]

Appendix B: Tables

Table 1: Simulation Results for the scenario where for Y_{11}: CTL=0,A1=-10,A2=-10 and Y_{12}: CTL=0,A1=0,A2=0. Under this scenario, the correct arm to retain during the arm-dropping analysis is A2. The feasibility and arm dropping analysis occurs once 300 and 150 outcomes have been obtained, respectively.

Condition	Arm Dropping Analysis				Feasibility Analysis	Phase III Analysis								Overall Operating Characteristics	
Printtable[, 1]	A1	A2	Both	P(Correct Decision)	P(Continue)	A2:A1:B1	A2:A1	A2:B1	A1:B1	A2.1	A1.1	B1	None	Power	Type I Error
A1 = 0, A2 = 0, B1 = 0	0.05(0.005)	0.94(0.005)	0(0.001)	0.94(0.005)	0.05(0.004)	0(0)	0(0)	0(0)	0(0)	0(0.001)	0(0)	0.05(0.004)	0.95(0.004)	NA(NA)	0.05(0.004)
A1 = 0, A2 = 0, B1 = -0.1	0.05(0.004)	0.95(0.005)	0(0.001)	0.95(0.005)	0.05(0.005)	0(0)	0(0)	0(0.001)	0(0)	0(0)	0(0)	0.89(0.006)	0.11(0.006)	0.89(0.006)	0(0.001)
A1 = 0.1, A2 = 0, B1 = 0	0.04(0.004)	0.95(0.004)	0(0.001)	0.95(0.004)	0.05(0.005)	0(0)	0(0)	0(0)	0(0)	0(0.001)	0(0.001)	0.05(0.004)	0.95(0.004)	0(0.001)	0.05(0.004)
A1 = 0.1, A2 = 0, B1 = -0.1	0.05(0.005)	0.95(0.005)	0(0.001)	0.95(0.005)	0.05(0.004)	0(0)	0(0)	0(0.001)	0(0.001)	0(0.001)	0(0)	0.89(0.006)	0.1(0.006)	0.89(0.006)	0(0.001)
A1 = 0, A2 = 0.1, B1 = 0	0.05(0.004)	0.95(0.004)	0(0.001)	0.95(0.004)	0.05(0.004)	0(0)	0(0)	0(0.001)	0(0)	0.03(0.003)	0(0)	0.05(0.004)	0.92(0.005)	0.03(0.003)	0.05(0.004)
A1 = 0, A2 = 0.1, B1 = -0.1	0.05(0.004)	0.95(0.004)	0(0.001)	0.95(0.004)	0.05(0.004)	0(0)	0(0)	0.03(0.003)	0(0)	0.01(0.001)	0(0)	0.85(0.007)	0.11(0.006)	0.89(0.006)	0(0)
A1 = 0.1, A2 = 0.1, B1 = 0	0.04(0.004)	0.95(0.004)	0(0.001)	0.95(0.004)	0.04(0.004)	0(0)	0(0)	0(0.001)	0(0)	0.03(0.003)	0(0.001)	0.05(0.004)	0.92(0.005)	0.03(0.003)	0.05(0.004)
A1 = 0.1, A2 = 0.1, B1 = -0.1	0.05(0.005)	0.95(0.005)	0(0.001)	0.95(0.005)	0.06(0.005)	0(0)	0(0)	0.04(0.004)	0(0.001)	0.01(0.002)	0(0)	0.83(0.008)	0.12(0.007)	0.88(0.007)	NA(NA)

Table 2: Simulation Results for the scenario where for Y_{11}: CTL=0,A1=-10,A2=0 and Y_{12}: CTL=0,A1=0,A2=0. Under this scenario, the correct arm to retain during the arm-dropping analysis is A1. The feasibility and arm dropping analysis occurs once 300 and 150 outcomes have been obtained, respectively.

Condition	Arm Dropping Analysis				Feasibility Analysis	Phase III Analysis								Overall Operating Characteristics	
Printtable[, 1]	A1	A2	Both	P(Correct Decision)	P(Continue)	A2:A1:B1	A2:A1	A2:B1	A1:B1	A2.1	A1.1	B1	None	Power	Type I Error
A1 = 0, A2 = 0, B1 = 0	0.56(0.01)	0.42(0.01)	0.02(0.003)	0.56(0.01)	0.05(0.004)	0(0)	0(0)	0(0)	0(0)	0(0.001)	0(0)	0.05(0.004)	0.95(0.004)	NA(NA)	0.05(0.004)
A1 = 0, A2 = 0, B1 = -0.1	0.58(0.01)	0.41(0.01)	0.01(0.002)	0.58(0.01)	0.05(0.004)	0(0)	0(0)	0(0.001)	0(0.001)	0(0)	0(0)	0.89(0.006)	0.1(0.006)	0.89(0.006)	0(0.001)
A1 = 0.1, A2 = 0, B1 = 0	0.56(0.01)	0.42(0.01)	0.02(0.003)	0.56(0.01)	0.05(0.004)	0(0)	0(0)	0(0)	0(0)	0(0.001)	0.02(0.003)	0.04(0.004)	0.93(0.005)	0.02(0.003)	0.05(0.004)
A1 = 0.1, A2 = 0, B1 = -0.1	0.56(0.01)	0.43(0.01)	0.02(0.002)	0.56(0.01)	0.05(0.004)	0(0)	0(0)	0(0)	0.02(0.003)	0(0)	0(0.001)	0.86(0.007)	0.11(0.006)	0.89(0.006)	0(0.001)
A1 = 0, A2 = 0.1, B1 = 0	0.56(0.01)	0.42(0.01)	0.01(0.002)	0.56(0.01)	0.05(0.004)	0(0)	0(0)	0(0.001)	0(0)	0.01(0.002)	0(0.001)	0.05(0.004)	0.93(0.005)	0.01(0.002)	0.05(0.004)
A1 = 0, A2 = 0.1, B1 = -0.1	0.58(0.01)	0.41(0.01)	0.01(0.002)	0.58(0.01)	0.05(0.005)	0(0)	0(0)	0.01(0.002)	0(0.001)	0(0.001)	0(0)	0.87(0.007)	0.12(0.006)	0.88(0.006)	0(0.001)
A1 = 0.1, A2 = 0.1, B1 = 0	0.57(0.01)	0.42(0.01)	0.01(0.002)	0.57(0.01)	0.05(0.004)	0(0)	0(0.001)	0(0)	0(0)	0.02(0.003)	0.02(0.003)	0.05(0.004)	0.92(0.006)	0.04(0.004)	0.05(0.004)
A1 = 0.1, A2 = 0.1, B1 = -0.1	0.56(0.01)	0.42(0.01)	0.01(0.002)	0.56(0.01)	0.05(0.004)	0(0.001)	0(0)	0.01(0.002)	0.02(0.003)	0(0.001)	0(0.001)	0.85(0.007)	0.11(0.006)	0.89(0.006)	NA(NA)

Table 3: Simulation Results for the scenario where for Y_{11}: CTL=0,A1=0,A2=-10 and Y_{12}: CTL=0,A1=0,A2=0. Under this scenario, the correct arm to retain during the arm-dropping analysis is A2. The feasibility and arm dropping analysis occurs once 300 and 150 outcomes have been obtained, respectively.

Condition	Arm Dropping Analysis				Feasibility Analysis	Phase III Analysis								Overall Operating Characteristics	
Printtable[, 1]	A1	A2	Both	P(Correct Decision)	P(Continue)	A2:A1:B1	A2:A1	A2:B1	A1:B1	A2.1	A1.1	B1	None	Power	Type I Error
A1 = 0, A2 = 0, B1 = 0	0.01(0.002)	0.97(0.003)	0.02(0.002)	0.97(0.003)	0.05(0.004)	0(0)	0(0)	0(0)	0(0)	0(0.001)	0(0)	0.05(0.004)	0.95(0.005)	NA(NA)	0.05(0.005)
A1 = 0, A2 = 0, B1 = -0.1	0.01(0.002)	0.97(0.003)	0.01(0.002)	0.97(0.003)	0.05(0.005)	0(0)	0(0)	0(0.001)	0(0)	0(0)	0(0)	0.89(0.006)	0.1(0.006)	0.89(0.006)	0(0.001)
A1 = 0.1, A2 = 0, B1 = 0	0.01(0.002)	0.98(0.003)	0.01(0.002)	0.98(0.003)	0.05(0.004)	0(0)	0(0)	0(0)	0(0)	0(0.001)	0(0.001)	0.05(0.004)	0.95(0.004)	0(0.001)	0.05(0.004)
A1 = 0.1, A2 = 0, B1 = -0.1	0.01(0.002)	0.98(0.003)	0.01(0.002)	0.98(0.003)	0.05(0.004)	0(0)	0(0)	0(0.001)	0(0)	0(0)	0(0)	0.9(0.006)	0.1(0.006)	0.9(0.006)	0(0.001)
A1 = 0, A2 = 0.1, B1 = 0	0.01(0.002)	0.98(0.003)	0.01(0.002)	0.98(0.003)	0.05(0.004)	0(0)	0(0)	0(0.001)	0(0)	0.03(0.003)	0(0)	0.04(0.004)	0.92(0.005)	0.03(0.003)	0.05(0.004)
A1 = 0, A2 = 0.1, B1 = -0.1	0.01(0.002)	0.98(0.003)	0.01(0.002)	0.98(0.003)	0.05(0.004)	0(0)	0(0)	0.03(0.004)	0(0)	0.01(0.001)	0(0)	0.83(0.007)	0.13(0.007)	0.87(0.007)	0(0)
A1 = 0.1, A2 = 0.1, B1 = 0	0.01(0.002)	0.98(0.003)	0.02(0.002)	0.98(0.003)	0.05(0.004)	0(0)	0(0)	0(0.001)	0(0)	0.04(0.004)	0(0.001)	0.05(0.004)	0.92(0.006)	0.04(0.004)	0.05(0.004)
A1 = 0.1, A2 = 0.1, B1 = -0.1	0.01(0.002)	0.98(0.003)	0.01(0.002)	0.98(0.003)	0.05(0.004)	0(0)	0(0)	0.03(0.003)	0(0.001)	0.01(0.002)	0(0)	0.84(0.007)	0.12(0.007)	0.88(0.007)	NA(NA)

Table 4: Simulation Results for the scenario where for Y_{11}: CTL=0,A1=0,A2=0 and Y_{12}: CTL=0,A1=0,A2=0. Under this scenario, the correct arm to retain during the arm-dropping analysis is A2. The feasibility and arm dropping analysis occurs once 300 and 150 outcomes have been obtained, respectively.

Condition	Arm Dropping Analysis				Feasibility Analysis	Phase III Analysis								Overall Operating Characteristics	
Printtable[, 1]	A1	A2	Both	P(Correct Decision)	P(Continue)	A2:A1:B1	A2:A1	A2:B1	A1:B1	A2.1	A1.1	B1	None	Power	Type I Error
A1 = 0, A2 = 0, B1 = 0	0.05(0.004)	0.95(0.004)	0(0.001)	0.95(0.004)	0.05(0.005)	0(0)	0(0)	0(0)	0(0)	0(0.001)	0(0)	0.05(0.004)	0.95(0.004)	NA(NA)	0.05(0.004)
A1 = 0, A2 = 0, B1 = -0.1	0.05(0.004)	0.95(0.004)	0(0.001)	0.95(0.004)	0.05(0.004)	0(0)	0(0)	0(0.001)	0(0)	0(0)	0(0)	0.89(0.006)	0.11(0.006)	0.89(0.006)	0(0.001)
A1 = 0.1, A2 = 0, B1 = 0	0.04(0.004)	0.95(0.004)	0(0)	0.95(0.004)	0.06(0.005)	0(0)	0(0)	0(0)	0(0)	0(0)	0(0.001)	0.05(0.004)	0.95(0.004)	0(0.001)	0.05(0.004)
A1 = 0.1, A2 = 0, B1 = -0.1	0.04(0.004)	0.96(0.004)	0(0.001)	0.96(0.004)	0.05(0.004)	0(0)	0(0)	0(0.001)	0(0.001)	0(0)	0(0.001)	0.88(0.007)	0.12(0.006)	0.88(0.006)	0(0.001)
A1 = 0, A2 = 0.1, B1 = 0	0.05(0.004)	0.95(0.004)	0(0.001)	0.95(0.004)	0.06(0.005)	0(0)	0(0)	0(0.001)	0(0)	0.04(0.004)	0(0)	0.06(0.005)	0.9(0.006)	0.04(0.004)	0.06(0.005)
A1 = 0, A2 = 0.1, B1 = -0.1	0.06(0.005)	0.94(0.005)	0(0.001)	0.94(0.005)	0.04(0.004)	0(0)	0(0)	0.03(0.003)	0(0)	0(0.001)	0(0)	0.85(0.007)	0.11(0.006)	0.89(0.006)	0(0)
A1 = 0.1, A2 = 0.1, B1 = 0	0.04(0.004)	0.95(0.004)	0(0.001)	0.95(0.004)	0.05(0.004)	0(0)	0(0)	0(0.001)	0(0)	0.04(0.004)	0(0.001)	0.04(0.004)	0.91(0.006)	0.04(0.004)	0.05(0.004)
A1 = 0.1, A2 = 0.1, B1 = -0.1	0.04(0.004)	0.96(0.004)	0(0.001)	0.96(0.004)	0.05(0.004)	0(0)	0(0)	0.04(0.004)	0(0.001)	0(0.001)	0(0)	0.83(0.007)	0.13(0.007)	0.87(0.007)	NA(NA)

Table 5: Simulation Results for the scenario where for Y_{11}: CTL=0,A1=-10,A2=-10 and Y_{12}: CTL=0,A1=0,A2=10. Under this scenario, the correct arm to retain during the arm-dropping analysis is A2. The feasibility and arm dropping analysis occurs once 300 and 150 outcomes have been obtained, respectively.

Condition	Arm Dropping Analysis				Feasibility Analysis	Phase III Analysis								Overall Operating Characteristics	
Printtable[, 1]	A1	A2	Both	P(Correct Decision)	P(Continue)	A2:A1:B1	A2:A1	A2:B1	A1:B1	A2.1	A1.1	B1	None	Power	Type I Error
A1 = 0, A2 = 0, B1 = 0	0.01(0.002)	0.97(0.004)	0.02(0.003)	0.97(0.004)	0.86(0.007)	0(0)	0(0)	0(0.001)	0(0)	0.02(0.003)	0(0)	0.03(0.003)	0.95(0.004)	NA(NA)	0.05(0.004)
A1 = 0, A2 = 0, B1 = -0.1	0.01(0.002)	0.98(0.003)	0.02(0.003)	0.98(0.003)	0.87(0.007)	0(0)	0(0)	0.04(0.004)	0(0)	0(0.001)	0(0)	0.8(0.008)	0.16(0.007)	0.8(0.008)	0.04(0.004)
A1 = 0.1, A2 = 0, B1 = 0	0.01(0.002)	0.98(0.003)	0.02(0.003)	0.98(0.003)	0.86(0.007)	0(0)	0(0)	0(0.001)	0(0)	0.02(0.003)	0.01(0.002)	0.02(0.003)	0.94(0.005)	0.01(0.002)	0.05(0.004)
A1 = 0.1, A2 = 0, B1 = -0.1	0.01(0.002)	0.97(0.003)	0.02(0.003)	0.97(0.003)	0.87(0.007)	0(0.001)	0(0)	0.05(0.004)	0.01(0.002)	0(0.001)	0(0.001)	0.78(0.008)	0.17(0.007)	0.78(0.008)	0.05(0.004)
A1 = 0, A2 = 0.1, B1 = 0	0.01(0.002)	0.97(0.003)	0.02(0.003)	0.97(0.003)	0.88(0.007)	0(0)	0(0)	0.03(0.004)	0(0)	0.55(0.01)	0(0)	0.01(0.002)	0.4(0.01)	0.55(0.01)	0.05(0.004)
A1 = 0, A2 = 0.1, B1 = -0.1	0.01(0.002)	0.98(0.003)	0.02(0.002)	0.98(0.003)	0.88(0.007)	0(0)	0(0)	0.64(0.01)	0(0)	0.08(0.005)	0(0)	0.25(0.009)	0.04(0.004)	0.96(0.004)	0(0)
A1 = 0.1, A2 = 0.1, B1 = 0	0.01(0.002)	0.98(0.003)	0.01(0.002)	0.98(0.003)	0.87(0.007)	0(0)	0(0.001)	0.04(0.004)	0(0)	0.63(0.01)	0(0.001)	0.01(0.002)	0.32(0.009)	0.63(0.01)	0.05(0.004)
A1 = 0.1, A2 = 0.1, B1 = -0.1	0.01(0.002)	0.97(0.003)	0.02(0.003)	0.97(0.003)	0.87(0.007)	0.01(0.002)	0(0)	0.66(0.009)	0(0.001)	0.09(0.006)	0(0)	0.2(0.008)	0.03(0.003)	0.97(0.003)	NA(NA)

Table 6: Simulation Results for the scenario where for Y_{11}: CTL=0,A1=-10,A2=0 and Y_{12}: CTL=0,A1=0,A2=10. Under this scenario, the correct arm to retain during the arm-dropping analysis is Both. The feasibility and arm dropping analysis occurs once 300 and 150 outcomes have been obtained, respectively.

Condition	Arm Dropping Analysis				Feasibility Analysis	Phase III Analysis								Overall Operating Characteristics	
Printtable[, 1]	A1	A2	Both	P(Correct Decision)	P(Continue)	A2:A1:B1	A2:A1	A2:B1	A1:B1	A2.1	A1.1	B1	None	Power	Type I Error
A1 = 0, A2 = 0, B1 = 0	0.15(0.007)	0.43(0.01)	0.41(0.01)	0.41(0.01)	0.66(0.01)	0(0)	0(0)	0(0.001)	0(0)	0.01(0.002)	0(0)	0.03(0.003)	0.96(0.004)	NA(NA)	0.04(0.004)
A1 = 0, A2 = 0, B1 = -0.1	0.17(0.007)	0.42(0.01)	0.42(0.01)	0.42(0.01)	0.65(0.01)	0(0.001)	0(0)	0.02(0.003)	0.01(0.001)	0(0.001)	0(0)	0.81(0.008)	0.17(0.007)	0.81(0.008)	0.03(0.003)
A1 = 0.1, A2 = 0, B1 = 0	0.15(0.007)	0.42(0.01)	0.43(0.01)	0.43(0.01)	0.66(0.009)	0(0.001)	0(0.001)	0(0)	0(0.001)	0.01(0.002)	0.15(0.007)	0.03(0.003)	0.81(0.008)	0.15(0.007)	0.05(0.004)
A1 = 0.1, A2 = 0, B1 = -0.1	0.14(0.007)	0.44(0.01)	0.42(0.01)	0.42(0.01)	0.66(0.009)	0.01(0.002)	0(0)	0.02(0.003)	0.15(0.007)	0(0.001)	0.03(0.004)	0.66(0.009)	0.12(0.007)	0.84(0.007)	0.04(0.004)
A1 = 0, A2 = 0.1, B1 = 0	0.14(0.007)	0.43(0.01)	0.43(0.01)	0.43(0.01)	0.66(0.009)	0(0.001)	0(0.001)	0.02(0.003)	0(0)	0.37(0.01)	0(0.001)	0.02(0.003)	0.58(0.01)	0.37(0.01)	0.05(0.004)
A1 = 0, A2 = 0.1, B1 = -0.1	0.16(0.007)	0.44(0.01)	0.4(0.01)	0.4(0.01)	0.65(0.01)	0(0.001)	0(0)	0.38(0.01)	0.01(0.001)	0.06(0.005)	0(0.001)	0.47(0.01)	0.08(0.005)	0.91(0.006)	0.01(0.002)
A1 = 0.1, A2 = 0.1, B1 = 0	0.15(0.007)	0.42(0.01)	0.43(0.01)	0.43(0.01)	0.65(0.01)	0.01(0.001)	0.09(0.006)	0.01(0.002)	0(0.001)	0.3(0.009)	0.05(0.005)	0.02(0.003)	0.51(0.01)	0.45(0.01)	0.04(0.004)
A1 = 0.1, A2 = 0.1, B1 = -0.1	0.16(0.007)	0.42(0.01)	0.41(0.01)	0.41(0.01)	0.64(0.01)	0.1(0.006)	0.01(0.002)	0.3(0.009)	0.05(0.005)	0.04(0.004)	0.01(0.002)	0.41(0.01)	0.06(0.005)	0.94(0.005)	NA(NA)

Table 7: Simulation Results for the scenario where for Y_{11}: CTL=0,A1=0,A2=-10 and Y_{12}: CTL=0,A1=0,A2=10. Under this scenario, the correct arm to retain during the arm-dropping analysis is A2. The feasibility and arm dropping analysis occurs once 300 and 150 outcomes have been obtained, respectively.

Condition	Arm Dropping Analysis				Feasibility Analysis	Phase III Analysis								Overall Operating Characteristics	
Printtable[, 1]	A1	A2	Both	P(Correct Decision)	P(Continue)	A2:A1:B1	A2:A1	A2:B1	A1:B1	A2.1	A1.1	B1	None	Power	Type I Error
A1 = 0, A2 = 0, B1 = 0	0(0)	1(0)	0(0)	1(0)	0.89(0.006)	0(0)	0(0)	0(0.001)	0(0)	0.02(0.003)	0(0)	0.02(0.003)	0.95(0.004)	NA(NA)	0.05(0.004)
A1 = 0, A2 = 0, B1 = -0.1	0(0)	1(0)	0(0)	1(0)	0.89(0.006)	0(0)	0(0)	0.04(0.004)	0(0)	0(0.001)	0(0)	0.8(0.008)	0.16(0.007)	0.8(0.008)	0.04(0.004)
A1 = 0.1, A2 = 0, B1 = 0	0(0)	1(0)	0(0)	1(0)	0.88(0.006)	0(0)	0(0)	0(0.001)	0(0)	0.03(0.003)	0(0)	0.03(0.003)	0.94(0.005)	0(0)	0.06(0.005)
A1 = 0.1, A2 = 0, B1 = -0.1	0(0)	1(0)	0(0)	1(0)	0.88(0.006)	0(0)	0(0)	0.05(0.004)	0(0)	0(0.001)	0(0)	0.79(0.008)	0.16(0.007)	0.79(0.008)	0.05(0.005)
A1 = 0, A2 = 0.1, B1 = 0	0(0)	1(0)	0(0)	1(0)	0.88(0.006)	0(0)	0(0)	0.04(0.004)	0(0)	0.55(0.01)	0(0)	0.02(0.002)	0.4(0.01)	0.55(0.01)	0.05(0.005)
A1 = 0, A2 = 0.1, B1 = -0.1	0(0)	1(0)	0(0)	1(0)	0.89(0.006)	0(0)	0(0)	0.65(0.01)	0(0)	0.08(0.005)	0(0)	0.24(0.009)	0.03(0.004)	0.97(0.004)	0(0)
A1 = 0.1, A2 = 0.1, B1 = 0	0(0)	1(0)	0(0)	1(0)	0.88(0.007)	0(0)	0(0)	0.04(0.004)	0(0)	0.64(0.01)	0(0)	0.01(0.002)	0.31(0.009)	0.64(0.01)	0.05(0.004)
A1 = 0.1, A2 = 0.1, B1 = -0.1	0(0)	1(0)	0(0)	1(0)	0.89(0.006)	0(0)	0(0)	0.67(0.009)	0(0)	0.1(0.006)	0(0)	0.19(0.008)	0.04(0.004)	0.96(0.004)	NA(NA)

Table 8: Simulation Results for the scenario where for Y_{11}: CTL=0,A1=0,A2=0 and Y_{12}: CTL=0,A1=0,A2=10. Under this scenario, the correct arm to retain during the arm-dropping analysis is A2. The feasibility and arm dropping analysis occurs once 300 and 150 outcomes have been obtained, respectively.

Condition	Arm Dropping Analysis				Feasibility Analysis	Phase III Analysis								Overall Operating Characteristics	
Printtable[, 1]	A1	A2	Both	P(Correct Decision)	P(Continue)	A2:A1:B1	A2:A1	A2:B1	A1:B1	A2.1	A1.1	B1	None	Power	Type I Error
A1 = 0, A2 = 0, B1 = 0	0.01(0.001)	0.97(0.003)	0.02(0.003)	0.97(0.003)	0.87(0.007)	0(0)	0(0)	0(0.001)	0(0)	0.02(0.003)	0(0)	0.02(0.003)	0.95(0.004)	NA(NA)	0.05(0.004)
A1 = 0, A2 = 0, B1 = -0.1	0(0.001)	0.98(0.003)	0.02(0.003)	0.98(0.003)	0.88(0.007)	0(0)	0(0)	0.04(0.004)	0(0)	0.01(0.001)	0(0)	0.78(0.008)	0.17(0.008)	0.78(0.008)	0.04(0.004)
A1 = 0.1, A2 = 0, B1 = 0	0.01(0.002)	0.97(0.003)	0.02(0.003)	0.97(0.003)	0.88(0.007)	0(0)	0(0)	0(0.001)	0(0)	0.03(0.003)	0.01(0.002)	0.02(0.003)	0.94(0.005)	0.01(0.002)	0.05(0.004)
A1 = 0.1, A2 = 0, B1 = -0.1	0.01(0.001)	0.97(0.003)	0.02(0.003)	0.97(0.003)	0.88(0.007)	0(0)	0(0)	0.04(0.004)	0.01(0.002)	0.01(0.002)	0(0.001)	0.79(0.008)	0.15(0.007)	0.8(0.008)	0.05(0.004)
A1 = 0, A2 = 0.1, B1 = 0	0.01(0.002)	0.97(0.003)	0.02(0.003)	0.97(0.003)	0.87(0.007)	0(0)	0(0)	0.03(0.004)	0(0)	0.55(0.01)	0(0)	0.01(0.002)	0.41(0.01)	0.55(0.01)	0.05(0.004)
A1 = 0, A2 = 0.1, B1 = -0.1	0.01(0.002)	0.97(0.003)	0.02(0.003)	0.97(0.003)	0.87(0.007)	0(0)	0(0)	0.6(0.01)	0(0)	0.09(0.006)	0(0)	0.26(0.009)	0.05(0.004)	0.95(0.004)	0(0)
A1 = 0.1, A2 = 0.1, B1 = 0	0(0.001)	0.98(0.003)	0.02(0.003)	0.98(0.003)	0.88(0.006)	0(0)	0.01(0.001)	0.03(0.004)	0(0)	0.63(0.01)	0(0.001)	0.01(0.002)	0.32(0.009)	0.63(0.01)	0.04(0.004)
A1 = 0.1, A2 = 0.1, B1 = -0.1	0.01(0.002)	0.97(0.003)	0.02(0.003)	0.97(0.003)	0.87(0.007)	0.01(0.002)	0(0)	0.66(0.009)	0(0.001)	0.09(0.006)	0(0)	0.21(0.008)	0.03(0.004)	0.97(0.004)	NA(NA)

Table 9: Simulation Results for the scenario where for Y_{11}: CTL=0,A1=-10,A2=-10 and Y_{12}: CTL=0,A1=10,A2=0. Under this scenario, the correct arm to retain during the arm-dropping analysis is A1. The feasibility and arm dropping analysis occurs once 300 and 150 outcomes have been obtained, respectively.

Condition	Arm Dropping Analysis				Feasibility Analysis	Phase III Analysis								Overall Operating Characteristics	
Printtable[, 1]	A1	A2	Both	P(Correct Decision)	P(Continue)	A2:A1:B1	A2:A1	A2:B1	A1:B1	A2.1	A1.1	B1	None	Power	Type I Error
A1 = 0, A2 = 0, B1 = 0	0.73(0.009)	0.25(0.009)	0.01(0.002)	0.73(0.009)	0.72(0.009)	0(0)	0(0)	0(0)	0(0.001)	0(0.001)	0.02(0.002)	0.03(0.003)	0.96(0.004)	NA(NA)	0.04(0.004)
A1 = 0, A2 = 0, B1 = -0.1	0.71(0.009)	0.27(0.009)	0.02(0.003)	0.71(0.009)	0.7(0.009)	0(0)	0(0)	0.01(0.001)	0.02(0.003)	0(0)	0(0.001)	0.82(0.008)	0.15(0.007)	0.82(0.008)	0.03(0.003)
A1 = 0.1, A2 = 0, B1 = 0	0.72(0.009)	0.27(0.009)	0.02(0.002)	0.72(0.009)	0.7(0.009)	0(0)	0(0)	0(0)	0.03(0.003)	0(0.001)	0.4(0.01)	0.02(0.003)	0.55(0.01)	0.4(0.01)	0.04(0.004)
A1 = 0.1, A2 = 0, B1 = -0.1	0.73(0.009)	0.25(0.009)	0.02(0.003)	0.73(0.009)	0.72(0.009)	0(0.001)	0(0)	0(0.001)	0.47(0.01)	0(0)	0.06(0.005)	0.4(0.01)	0.07(0.005)	0.93(0.005)	0(0.001)
A1 = 0, A2 = 0.1, B1 = 0	0.72(0.009)	0.26(0.009)	0.02(0.003)	0.72(0.009)	0.71(0.009)	0(0)	0(0)	0(0.001)	0(0.001)	0.04(0.004)	0.01(0.002)	0.03(0.003)	0.91(0.006)	0.04(0.004)	0.05(0.004)
A1 = 0, A2 = 0.1, B1 = -0.1	0.73(0.009)	0.25(0.009)	0.01(0.002)	0.73(0.009)	0.73(0.009)	0(0)	0(0)	0.05(0.005)	0.03(0.004)	0(0.001)	0.01(0.001)	0.75(0.009)	0.15(0.007)	0.81(0.008)	0.04(0.004)
A1 = 0.1, A2 = 0.1, B1 = 0	0.71(0.009)	0.28(0.009)	0.02(0.003)	0.71(0.009)	0.7(0.009)	0(0.001)	0(0.001)	0(0.001)	0.03(0.003)	0.05(0.004)	0.45(0.01)	0.02(0.003)	0.45(0.01)	0.5(0.01)	0.05(0.004)
A1 = 0.1, A2 = 0.1, B1 = -0.1	0.74(0.009)	0.24(0.009)	0.02(0.003)	0.74(0.009)	0.73(0.009)	0(0.001)	0(0)	0.05(0.004)	0.51(0.01)	0.01(0.002)	0.07(0.005)	0.32(0.009)	0.04(0.004)	0.96(0.004)	NA(NA)

Table 10: Simulation Results for the scenario where for Y_{11}: CTL=0,A1=-10,A2=0 and Y_{12}: CTL=0,A1=10,A2=0. Under this scenario, the correct arm to retain during the arm-dropping analysis is A1. The feasibility and arm dropping analysis occurs once 300 and 150 outcomes have been obtained, respectively.

Condition	Arm Dropping Analysis				Feasibility Analysis	Phase III Analysis								Overall Operating Characteristics	
Printtable[, 1]	A1	A2	Both	P(Correct Decision)	P(Continue)	A2:A1:B1	A2:A1	A2:B1	A1:B1	A2.1	A1.1	B1	None	Power	Type I Error
A1 = 0, A2 = 0, B1 = 0	0.89(0.006)	0.11(0.006)	0(0)	0.89(0.006)	0.82(0.008)	0(0)	0(0)	0(0)	0(0.001)	0(0.001)	0.02(0.002)	0.03(0.003)	0.95(0.004)	NA(NA)	0.05(0.004)
A1 = 0, A2 = 0, B1 = -0.1	0.88(0.006)	0.12(0.006)	0(0)	0.88(0.006)	0.81(0.008)	0(0)	0(0)	0(0.001)	0.04(0.004)	0(0)	0.01(0.001)	0.81(0.008)	0.14(0.007)	0.81(0.008)	0.05(0.004)
A1 = 0.1, A2 = 0, B1 = 0	0.9(0.006)	0.1(0.006)	0(0)	0.9(0.006)	0.82(0.008)	0(0)	0(0)	0(0)	0.03(0.004)	0(0.001)	0.51(0.01)	0.02(0.002)	0.44(0.01)	0.51(0.01)	0.05(0.004)
A1 = 0.1, A2 = 0, B1 = -0.1	0.88(0.007)	0.12(0.007)	0(0)	0.88(0.007)	0.81(0.008)	0(0)	0(0)	0(0.001)	0.54(0.01)	0(0)	0.07(0.005)	0.34(0.009)	0.05(0.004)	0.95(0.004)	0(0.001)
A1 = 0, A2 = 0.1, B1 = 0	0.89(0.006)	0.11(0.006)	0(0)	0.89(0.006)	0.81(0.008)	0(0)	0(0)	0(0.001)	0(0)	0.01(0.002)	0.02(0.003)	0.02(0.003)	0.94(0.005)	0.01(0.002)	0.05(0.004)
A1 = 0, A2 = 0.1, B1 = -0.1	0.87(0.007)	0.13(0.007)	0(0)	0.87(0.007)	0.81(0.008)	0(0)	0(0)	0.02(0.003)	0.05(0.004)	0(0.001)	0(0.001)	0.77(0.008)	0.15(0.007)	0.8(0.008)	0.05(0.004)
A1 = 0.1, A2 = 0.1, B1 = 0	0.88(0.006)	0.12(0.006)	0(0)	0.88(0.006)	0.81(0.008)	0(0)	0(0)	0(0.001)	0.03(0.004)	0.02(0.003)	0.56(0.01)	0.01(0.002)	0.37(0.01)	0.58(0.01)	0.05(0.004)
A1 = 0.1, A2 = 0.1, B1 = -0.1	0.88(0.006)	0.12(0.006)	0(0)	0.88(0.006)	0.81(0.008)	0(0)	0(0)	0.03(0.003)	0.58(0.01)	0(0.001)	0.09(0.006)	0.26(0.009)	0.04(0.004)	0.96(0.004)	NA(NA)

Table 11: Simulation Results for the scenario where for Y_{11}: CTL=0,A1=0,A2=-10 and Y_{12}: CTL=0,A1=10,A2=0. Under this scenario, the correct arm to retain during the arm-dropping analysis is Both. The feasibility and arm dropping analysis occurs once 300 and 150 outcomes have been obtained, respectively.

Condition	Arm Dropping Analysis				Feasibility Analysis	Phase III Analysis								Overall Operating Characteristics	
Printtable[, 1]	A1	A2	Both	P(Correct Decision)	P(Continue)	A2:A1:B1	A2:A1	A2:B1	A1:B1	A2.1	A1.1	B1	None	Power	Type I Error
A1 = 0, A2 = 0, B1 = 0	0.32(0.009)	0.27(0.009)	0.42(0.01)	0.42(0.01)	0.59(0.01)	0(0)	0(0)	0(0)	0(0.001)	0.01(0.001)	0.01(0.002)	0.02(0.003)	0.97(0.004)	NA(NA)	0.03(0.004)
A1 = 0, A2 = 0, B1 = -0.1	0.31(0.009)	0.28(0.009)	0.41(0.01)	0.41(0.01)	0.57(0.01)	0(0.001)	0(0)	0(0.001)	0.01(0.002)	0(0.001)	0(0.001)	0.83(0.008)	0.15(0.007)	0.83(0.008)	0.02(0.003)
A1 = 0.1, A2 = 0, B1 = 0	0.31(0.009)	0.27(0.009)	0.42(0.01)	0.42(0.01)	0.58(0.01)	0(0.001)	0(0.001)	0(0)	0.01(0.002)	0(0.001)	0.29(0.009)	0.03(0.003)	0.66(0.009)	0.29(0.009)	0.05(0.004)
A1 = 0.1, A2 = 0, B1 = -0.1	0.32(0.009)	0.26(0.009)	0.41(0.01)	0.41(0.01)	0.59(0.01)	0.01(0.002)	0(0.001)	0.01(0.002)	0.33(0.009)	0(0.001)	0.05(0.005)	0.51(0.01)	0.08(0.006)	0.9(0.006)	0.02(0.003)
A1 = 0, A2 = 0.1, B1 = 0	0.31(0.009)	0.26(0.009)	0.43(0.01)	0.43(0.01)	0.59(0.01)	0(0)	0(0.001)	0.01(0.002)	0(0.001)	0.17(0.007)	0.01(0.002)	0.03(0.003)	0.78(0.008)	0.17(0.007)	0.05(0.004)
A1 = 0, A2 = 0.1, B1 = -0.1	0.31(0.009)	0.27(0.009)	0.42(0.01)	0.42(0.01)	0.56(0.01)	0.01(0.002)	0(0.001)	0.16(0.007)	0.02(0.003)	0.03(0.003)	0(0.001)	0.67(0.009)	0.11(0.006)	0.87(0.007)	0.03(0.003)
A1 = 0.1, A2 = 0.1, B1 = 0	0.32(0.009)	0.26(0.009)	0.42(0.01)	0.42(0.01)	0.57(0.01)	0.01(0.002)	0.09(0.006)	0(0.001)	0.01(0.002)	0.07(0.005)	0.24(0.008)	0.02(0.003)	0.57(0.01)	0.39(0.01)	0.05(0.004)
A1 = 0.1, A2 = 0.1, B1 = -0.1	0.32(0.009)	0.27(0.009)	0.41(0.01)	0.41(0.01)	0.58(0.01)	0.13(0.007)	0.02(0.002)	0.07(0.005)	0.24(0.008)	0.01(0.002)	0.03(0.003)	0.45(0.01)	0.07(0.005)	0.93(0.005)	NA(NA)

Table 12: Simulation Results for the scenario where for Y_{11}: CTL=0,A1=0,A2=0 and Y_{12}: CTL=0,A1=10,A2=0. Under this scenario, the correct arm to retain during the arm-dropping analysis is A1. The feasibility and arm dropping analysis occurs once 300 and 150 outcomes have been obtained, respectively.

Condition	Arm Dropping Analysis				Feasibility Analysis	Phase III Analysis								Overall Operating Characteristics	
Printtable[, 1]	A1	A2	Both	P(Correct Decision)	P(Continue)	A2:A1:B1	A2:A1	A2:B1	A1:B1	A2.1	A1.1	B1	None	Power	Type I Error
A1 = 0, A2 = 0, B1 = 0	0.71(0.009)	0.28(0.009)	0.02(0.003)	0.71(0.009)	0.69(0.009)	0(0)	0(0)	0(0)	0(0.001)	0(0.001)	0.02(0.003)	0.03(0.003)	0.95(0.004)	NA(NA)	0.05(0.004)
A1 = 0, A2 = 0, B1 = -0.1	0.72(0.009)	0.26(0.009)	0.02(0.002)	0.72(0.009)	0.7(0.009)	0(0)	0(0)	0(0.001)	0.03(0.003)	0(0.001)	0(0.001)	0.81(0.008)	0.16(0.007)	0.81(0.008)	0.03(0.004)
A1 = 0.1, A2 = 0, B1 = 0	0.72(0.009)	0.26(0.009)	0.02(0.003)	0.72(0.009)	0.72(0.009)	0(0)	0(0)	0(0)	0.03(0.003)	0(0.001)	0.42(0.01)	0.02(0.003)	0.53(0.01)	0.42(0.01)	0.05(0.004)
A1 = 0.1, A2 = 0, B1 = -0.1	0.73(0.009)	0.25(0.009)	0.02(0.003)	0.73(0.009)	0.71(0.009)	0(0)	0(0)	0(0.001)	0.48(0.01)	0(0)	0.06(0.005)	0.4(0.01)	0.06(0.005)	0.94(0.005)	0(0.001)
A1 = 0, A2 = 0.1, B1 = 0	0.73(0.009)	0.25(0.009)	0.02(0.003)	0.73(0.009)	0.73(0.009)	0(0)	0(0)	0(0.001)	0(0.001)	0.04(0.004)	0.02(0.003)	0.03(0.003)	0.9(0.006)	0.04(0.004)	0.06(0.005)
A1 = 0, A2 = 0.1, B1 = -0.1	0.72(0.009)	0.27(0.009)	0.01(0.002)	0.72(0.009)	0.7(0.009)	0(0)	0(0)	0.05(0.004)	0.03(0.004)	0.01(0.002)	0(0.001)	0.76(0.009)	0.15(0.007)	0.81(0.008)	0.04(0.004)
A1 = 0.1, A2 = 0.1, B1 = 0	0.74(0.009)	0.25(0.009)	0.01(0.002)	0.74(0.009)	0.72(0.009)	0(0)	0(0.001)	0(0.001)	0.02(0.003)	0.04(0.004)	0.46(0.01)	0.01(0.002)	0.45(0.01)	0.51(0.01)	0.04(0.004)
A1 = 0.1, A2 = 0.1, B1 = -0.1	0.72(0.009)	0.26(0.009)	0.02(0.003)	0.72(0.009)	0.71(0.009)	0.01(0.002)	0(0.001)	0.04(0.004)	0.49(0.01)	0.01(0.002)	0.07(0.005)	0.33(0.009)	0.05(0.004)	0.95(0.004)	NA(NA)

Table 13: Simulation Results for the scenario where for Y_{11}: CTL=0,A1=-10,A2=-10 and Y_{12}: CTL=0,A1=10,A2=10. Under this scenario, the correct arm to retain during the arm-dropping analysis is A2. The feasibility and arm dropping analysis occurs once 300 and 150 outcomes have been obtained, respectively.

Condition	Arm Dropping Analysis				Feasibility Analysis	Phase III Analysis								Overall Operating Characteristics	
Printtable[, 1]	A1	A2	Both	P(Correct Decision)	P(Continue)	A2:A1:B1	A2:A1	A2:B1	A1:B1	A2.1	A1.1	B1	None	Power	Type I Error
A1 = 0, A2 = 0, B1 = 0	0.05(0.004)	0.95(0.004)	0(0)	0.95(0.004)	0.99(0.002)	0(0)	0(0)	0(0.001)	0(0.001)	0.02(0.003)	0(0)	0.02(0.003)	0.95(0.004)	NA(NA)	0.05(0.004)
A1 = 0, A2 = 0, B1 = -0.1	0.05(0.004)	0.95(0.005)	0(0.001)	0.95(0.005)	0.99(0.002)	0(0)	0(0)	0.04(0.004)	0(0.001)	0(0.001)	0(0)	0.78(0.008)	0.18(0.008)	0.78(0.008)	0.05(0.004)
A1 = 0.1, A2 = 0, B1 = 0	0.05(0.004)	0.95(0.004)	0(0.001)	0.95(0.004)	1(0.001)	0(0)	0(0)	0(0.001)	0(0.001)	0.02(0.003)	0.03(0.004)	0.02(0.003)	0.92(0.005)	0.03(0.004)	0.05(0.004)
A1 = 0.1, A2 = 0, B1 = -0.1	0.06(0.005)	0.94(0.005)	0(0.001)	0.94(0.005)	0.99(0.002)	0(0)	0(0)	0.05(0.004)	0.04(0.004)	0.01(0.001)	0.01(0.001)	0.74(0.009)	0.16(0.007)	0.78(0.008)	0.06(0.005)
A1 = 0, A2 = 0.1, B1 = 0	0.05(0.004)	0.95(0.004)	0(0.001)	0.95(0.004)	0.99(0.001)	0(0)	0(0)	0.03(0.004)	0(0)	0.61(0.01)	0(0.001)	0.01(0.002)	0.35(0.01)	0.61(0.01)	0.04(0.004)
A1 = 0, A2 = 0.1, B1 = -0.1	0.05(0.004)	0.95(0.004)	0(0.001)	0.95(0.004)	0.99(0.001)	0(0)	0(0)	0.68(0.009)	0(0.001)	0.08(0.005)	0(0)	0.2(0.008)	0.04(0.004)	0.96(0.004)	0(0.001)
A1 = 0.1, A2 = 0.1, B1 = 0	0.05(0.004)	0.95(0.004)	0(0.001)	0.95(0.004)	1(0.001)	0(0)	0(0)	0.05(0.004)	0(0.001)	0.69(0.009)	0.04(0.004)	0.01(0.002)	0.21(0.008)	0.73(0.009)	0.06(0.005)
A1 = 0.1, A2 = 0.1, B1 = -0.1	0.05(0.004)	0.95(0.005)	0(0.001)	0.95(0.005)	0.99(0.002)	0(0.001)	0(0)	0.73(0.009)	0.04(0.004)	0.1(0.006)	0.01(0.002)	0.11(0.006)	0.02(0.003)	0.98(0.003)	NA(NA)

Table 14: Simulation Results for the scenario where for Y_{11}: CTL=0,A1=-10,A2=0 and Y_{12}: CTL=0,A1=10,A2=10. Under this scenario, the correct arm to retain during the arm-dropping analysis is A1. The feasibility and arm dropping analysis occurs once 300 and 150 outcomes have been obtained, respectively.

Condition	Arm Dropping Analysis				Feasibility Analysis	Phase III Analysis								Overall Operating Characteristics	
Printtable[, 1]	A1	A2	Both	P(Correct Decision)	P(Continue)	A2:A1:B1	A2:A1	A2:B1	A1:B1	A2.1	A1.1	B1	None	Power	Type I Error
A1 = 0, A2 = 0, B1 = 0	0.57(0.01)	0.41(0.01)	0.02(0.003)	0.57(0.01)	0.99(0.001)	0(0)	0(0)	0(0.001)	0(0.001)	0.01(0.002)	0.01(0.002)	0.02(0.003)	0.95(0.004)	NA(NA)	0.05(0.004)
A1 = 0, A2 = 0, B1 = -0.1	0.57(0.01)	0.41(0.01)	0.02(0.003)	0.57(0.01)	1(0.001)	0(0)	0(0)	0.02(0.003)	0.03(0.003)	0(0.001)	0(0.001)	0.78(0.008)	0.17(0.008)	0.78(0.008)	0.05(0.004)
A1 = 0.1, A2 = 0, B1 = 0	0.56(0.01)	0.42(0.01)	0.01(0.002)	0.56(0.01)	0.99(0.002)	0(0)	0(0)	0(0.001)	0.02(0.003)	0.01(0.002)	0.36(0.01)	0.01(0.002)	0.59(0.01)	0.36(0.01)	0.05(0.004)
A1 = 0.1, A2 = 0, B1 = -0.1	0.59(0.01)	0.4(0.01)	0.01(0.002)	0.59(0.01)	0.99(0.002)	0(0)	0(0)	0.02(0.003)	0.42(0.01)	0(0.001)	0.06(0.005)	0.41(0.01)	0.09(0.006)	0.89(0.006)	0.02(0.003)
A1 = 0, A2 = 0.1, B1 = 0	0.56(0.01)	0.42(0.01)	0.01(0.002)	0.56(0.01)	0.99(0.002)	0(0)	0(0)	0.02(0.003)	0(0.001)	0.27(0.009)	0.01(0.002)	0.02(0.003)	0.68(0.009)	0.27(0.009)	0.05(0.005)
A1 = 0, A2 = 0.1, B1 = -0.1	0.55(0.01)	0.43(0.01)	0.02(0.003)	0.55(0.01)	0.99(0.002)	0(0)	0(0)	0.31(0.009)	0.04(0.004)	0.05(0.004)	0(0.001)	0.5(0.01)	0.1(0.006)	0.86(0.007)	0.04(0.004)
A1 = 0.1, A2 = 0.1, B1 = 0	0.55(0.01)	0.43(0.01)	0.02(0.003)	0.55(0.01)	0.99(0.001)	0(0)	0.01(0.001)	0.02(0.003)	0.02(0.003)	0.32(0.009)	0.41(0.01)	0.01(0.002)	0.22(0.008)	0.73(0.009)	0.05(0.004)
A1 = 0.1, A2 = 0.1, B1 = -0.1	0.56(0.01)	0.42(0.01)	0.02(0.003)	0.56(0.01)	0.99(0.001)	0.01(0.002)	0(0.001)	0.33(0.009)	0.43(0.01)	0.04(0.004)	0.05(0.005)	0.11(0.006)	0.02(0.003)	0.98(0.003)	NA(NA)

Table 15: Simulation Results for the scenario where for Y_{11}: CTL=0,A1=0,A2=-10 and Y_{12}: CTL=0,A1=10,A2=10. Under this scenario, the correct arm to retain during the arm-dropping analysis is A2. The feasibility and arm dropping analysis occurs once 300 and 150 outcomes have been obtained, respectively.

Condition	Arm Dropping Analysis				Feasibility Analysis	Phase III Analysis								Overall Operating Characteristics	
Printtable[, 1]	A1	A2	Both	P(Correct Decision)	P(Continue)	A2:A1:B1	A2:A1	A2:B1	A1:B1	A2.1	A1.1	B1	None	Power	Type I Error
A1 = 0, A2 = 0, B1 = 0	0.02(0.002)	0.97(0.003)	0.02(0.002)	0.97(0.003)	0.99(0.001)	0(0)	0(0)	0(0.001)	0(0)	0.02(0.003)	0(0)	0.02(0.003)	0.95(0.004)	NA(NA)	0.05(0.004)
A1 = 0, A2 = 0, B1 = -0.1	0.01(0.002)	0.97(0.003)	0.02(0.002)	0.97(0.003)	1(0.001)	0(0)	0(0)	0.04(0.004)	0(0.001)	0.01(0.001)	0(0)	0.78(0.008)	0.17(0.007)	0.78(0.008)	0.05(0.004)
A1 = 0.1, A2 = 0, B1 = 0	0.01(0.002)	0.98(0.003)	0.01(0.002)	0.98(0.003)	0.99(0.001)	0(0)	0(0)	0(0.001)	0(0)	0.02(0.003)	0.01(0.002)	0.03(0.003)	0.94(0.005)	0.01(0.002)	0.06(0.005)
A1 = 0.1, A2 = 0, B1 = -0.1	0.01(0.002)	0.97(0.003)	0.02(0.003)	0.97(0.003)	0.99(0.001)	0(0)	0(0)	0.05(0.004)	0.02(0.003)	0(0.001)	0(0.001)	0.76(0.009)	0.17(0.007)	0.78(0.008)	0.05(0.004)
A1 = 0, A2 = 0.1, B1 = 0	0.01(0.002)	0.98(0.003)	0.02(0.003)	0.98(0.003)	1(0.001)	0(0)	0(0)	0.04(0.004)	0(0)	0.62(0.01)	0(0)	0.01(0.002)	0.33(0.009)	0.62(0.01)	0.05(0.004)
A1 = 0, A2 = 0.1, B1 = -0.1	0.01(0.002)	0.97(0.003)	0.02(0.003)	0.97(0.003)	0.99(0.001)	0(0.001)	0(0)	0.69(0.009)	0(0.001)	0.08(0.005)	0(0)	0.19(0.008)	0.04(0.004)	0.96(0.004)	0(0.001)
A1 = 0.1, A2 = 0.1, B1 = 0	0.01(0.002)	0.98(0.003)	0.01(0.002)	0.98(0.003)	0.99(0.001)	0(0)	0(0.001)	0.05(0.004)	0(0)	0.69(0.009)	0.01(0.002)	0.01(0.002)	0.24(0.009)	0.71(0.009)	0.05(0.004)
A1 = 0.1, A2 = 0.1, B1 = -0.1	0.01(0.002)	0.97(0.003)	0.02(0.002)	0.97(0.003)	1(0.001)	0.01(0.002)	0(0.001)	0.75(0.009)	0.01(0.002)	0.1(0.006)	0(0)	0.11(0.006)	0.02(0.003)	0.98(0.003)	NA(NA)

Table 16: Simulation Results for the scenario where for Y_{11}: CTL=0,A1=0,A2=0 and Y_{12}: CTL=0,A1=10,A2=10. Under this scenario, the correct arm to retain during the arm-dropping analysis is A2. The feasibility and arm dropping analysis occurs once 300 and 150 outcomes have been obtained, respectively.

Condition	Arm Dropping Analysis				Feasibility Analysis	Phase III Analysis								Overall Operating Characteristics	
Printtable[, 1]	A1	A2	Both	P(Correct Decision)	P(Continue)	A2:A1:B1	A2:A1	A2:B1	A1:B1	A2.1	A1.1	B1	None	Power	Type I Error
A1 = 0, A2 = 0, B1 = 0	0.04(0.004)	0.96(0.004)	0(0.001)	0.96(0.004)	0.99(0.001)	0(0)	0(0)	0.01(0.001)	0(0)	0.02(0.003)	0(0.001)	0.02(0.003)	0.95(0.004)	NA(NA)	0.05(0.004)
A1 = 0, A2 = 0, B1 = -0.1	0.05(0.004)	0.95(0.004)	0(0.001)	0.95(0.004)	0.99(0.002)	0(0)	0(0)	0.05(0.004)	0(0)	0(0.001)	0(0.001)	0.78(0.008)	0.17(0.008)	0.78(0.008)	0.05(0.004)
A1 = 0.1, A2 = 0, B1 = 0	0.05(0.004)	0.95(0.004)	0(0.001)	0.95(0.004)	0.99(0.002)	0(0)	0(0)	0(0.001)	0(0.001)	0.02(0.003)	0.03(0.003)	0.02(0.003)	0.93(0.005)	0.03(0.003)	0.04(0.004)
A1 = 0.1, A2 = 0, B1 = -0.1	0.05(0.004)	0.95(0.004)	0(0.001)	0.95(0.004)	0.99(0.001)	0(0)	0(0)	0.05(0.004)	0.04(0.004)	0.01(0.001)	0.01(0.001)	0.73(0.009)	0.17(0.008)	0.77(0.008)	0.06(0.005)
A1 = 0, A2 = 0.1, B1 = 0	0.05(0.004)	0.95(0.004)	0(0.001)	0.95(0.004)	0.99(0.002)	0(0)	0(0)	0.04(0.004)	0(0)	0.6(0.01)	0(0.001)	0.01(0.002)	0.35(0.01)	0.6(0.01)	0.05(0.004)
A1 = 0, A2 = 0.1, B1 = -0.1	0.05(0.004)	0.95(0.004)	0(0)	0.95(0.004)	0.99(0.001)	0(0)	0(0)	0.67(0.009)	0(0.001)	0.1(0.006)	0(0)	0.19(0.008)	0.03(0.004)	0.96(0.004)	0(0.001)
A1 = 0.1, A2 = 0.1, B1 = 0	0.05(0.004)	0.95(0.004)	0(0.001)	0.95(0.004)	1(0.001)	0(0)	0(0.001)	0.04(0.004)	0(0.001)	0.68(0.009)	0.03(0.003)	0.01(0.002)	0.23(0.008)	0.72(0.009)	0.05(0.004)
A1 = 0.1, A2 = 0.1, B1 = -0.1	0.04(0.004)	0.96(0.004)	0(0.001)	0.96(0.004)	1(0.001)	0(0)	0(0)	0.75(0.009)	0.03(0.004)	0.09(0.006)	0(0.001)	0.11(0.006)	0.02(0.003)	0.98(0.003)	NA(NA)
